# Lipid–polymer hybrid-vesicles interrupt nucleation of amyloid fibrillation†‡

**DOI:** 10.1039/d4cb00217b

**Published:** 2024-10-24

**Authors:** Newton Sen, Stephanie Krüger, Wolfgang H. Binder

**Affiliations:** a Macromolecular Chemistry, Institute of Chemistry, Faculty of Natural Science II (Chemistry, Physics and Mathematics), Martin Luther University Halle-Wittenberg von-Danckelmann-Platz 4 Halle D-06120 Germany wolfgang.binder@chemie.uni-halle.de; b Biocenter, Martin-Luther University Halle-Wittenberg Weinbergweg 22 Halle (Saale) D-06120 Germany

## Abstract

Solubility and aggregation of proteins are crucial factors for their functional and further biological roles. Aggregation of proteins *in vivo*, such as the amyloid beta (Aβ_1–40_) peptide into fibrils, is significantly modulated by membrane lipids, abundantly present in cells. We developed a model membrane system, composed of lipid hybrid-vesicles bearing embedded hydrophilic polymers to *in vitro* study the aggregation of the Aβ_1–40_ peptide. Focus is to understand and inhibit the primordial, nucleation stages of their fibrillation by added hybrid-vesicles, composed of a natural lipid and amphiphilic polymers. These designed hybrid-vesicles are based on 1-palmitoyl-2-oleoyl-glycero-3-phosphocholine (POPC), displaying embedded hydrophilic (EO)_*m*_P_*n*_A_**EG** polymers (*m* = 2 or 3; P_*n*_ = 10 to 52 with *M*_n_ = 2800–9950 gmol^−1^) in amounts ranging from 5–20 mol%, anchored to the POPC vesicles *via* hydrophobic hexadecyl-, glyceryl- and cholesteryl-moieties, affixed to the polymers as end-groups. All investigated hybrid-vesicles significantly delay fibrillation of the Aβ_1–40_ peptide as determined by thioflavin T (ThT) assays. We observed that the hybrid-vesicles interacted with early aggregating species of Aβ_1–40_ peptide, irrespective of their composition or size. A substantial perturbation of both primary (*k*_+_*k*_*n*_) and secondary (*k*_+_*k*_2_) nucleation rates of Aβ_1–40_ by the POPC–polymer vesicles compared to POPC vesicles was observed, particularly for the cholesteryl-anchored polymers, interfering with the fragmentation and elongation steps of Aβ_1–40_. Furthermore, morphological differences of the aggregates were revealed by transmission electron microscopy (TEM) images supported the inhibitory kinetic signatures.

## Abstract 2

Hybrid-vesicles composed of amphiphilic polymers in POPC-lipids inhibit Aβ_1–40_ peptide fibrillation by interfering with primary and secondary nucleation, most pronounced by the cholesteryl-anchored polymers.

## Graphical abstract

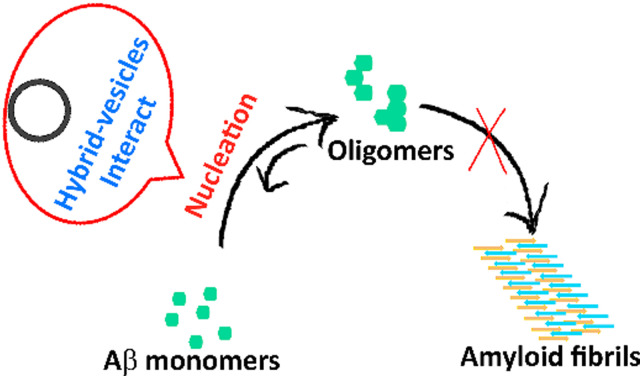

## Introduction

In humans, about 20, 000 distinct proteins are associated with the protein homeostasis,^1^ while the pathogenicity of amyloids is linked with over 50 various proteins and peptides.^2^ The pathogenicity of proteins manifests through the formation of highly ordered amyloid fibrillar structures, a densely packed cross β-strand stabilized by ‘steric zippers’, arising from the variable primary sequences of proteins which ultimately succumb to the formation of amyloid aggregate.^3^ These fibrillary forms of protein aggregation are involved in neurodegenerative diseases (Alzheimer's, Parkinson's)^4^ to insulin aggregation^5^ and eye lens protein aggregation.^6^

The non-covalent polymerization of soluble proteins into solid amyloids can be initiated by a reversible liquid–liquid phase transition, which then irreversibly can lead to the formation of solid amyloid condensates. When the monomer concentration reaches supersaturation in amyloidogenesis,^7,8^ their polymerization involves a cascade of microscopic steps like homo- and heterogeneous primary nucleation, secondary nucleation (small aggregates dependent), fragmentation, and elongation to insoluble fibrillar solids. Secondary nucleation, catalytic in nature,^9^ and the subsequent microscopic steps in a single process required for amyloidosis, follow classical and non-classical nucleation theory to connect microscopic steps kinetically and thermodynamically.^7,10,11^ To deeper understand the complexity of those aggregation processes, model systems (*in vitro*) are important to understand, modulate, and if possible, control this aggregation process, finally aiming for its inhibition. Thus the amyloidogenesis of amyloid beta (Aβ_1–40/42_) peptides is intricately regulated by various factors, both often intracellular (like pH, ions) and extracellular (like air–water interfaces, lipid membranes, other proteins like α-synuclein,^12^ preformed fibrils, small aggregates, seed concentrations and temperature) with the precise role of each factor being largely unclear,^13^ in view of the complex role in Alzheimer's disease pathogenicity.^14^ Neuronal biomembranes, where amyloid aggregation takes place, are mainly composed of phospholipids (both zwitterionic and anionic), cholesterol, ganglioside (GM1),^15,16^ and sphingomyelin,^17^ all potentially involved in the first steps of this complex protein assembly process. *In vitro* studies of physiochemical properties of lipid bilayers like membrane's phase, thickness,^18^ curvature,^19^ oxidative stress,^20–22^ among many others, produce a significant impact on amyloid aggregation.^23^ The use of lipid model systems, such as lipid vesicles composed of natural lipids like zwitterionic lipids (POPC, DMPC, and POPE), anionic lipids (POPS, POPG), neutral lipids (DOPC), cholesterol^24–26^ or a mixture of lipids offers a powerful platform to decipher the membrane role on Aβ_1–40/42_ fibrillation *in vitro*.^27,28^ Early stages of fibrillation, such as nucleation (primary, secondary, and surface-mediated nucleation) are profoundly decided by model lipids^14,29,30^ that can induce both, acceleration or inhibition of the fibrillation.^14,28,31–36^ In this context, efforts are directed towards targeting the deleterious early forms (oligomers, fibrillar segments, small liquid condensates) of the amyloid transformation to reduce amyloid beta peptide (Aβ_1–40/42_) pathogenicity by inhibition of early nucleation stages.^37,38^

**Fig. 1:**
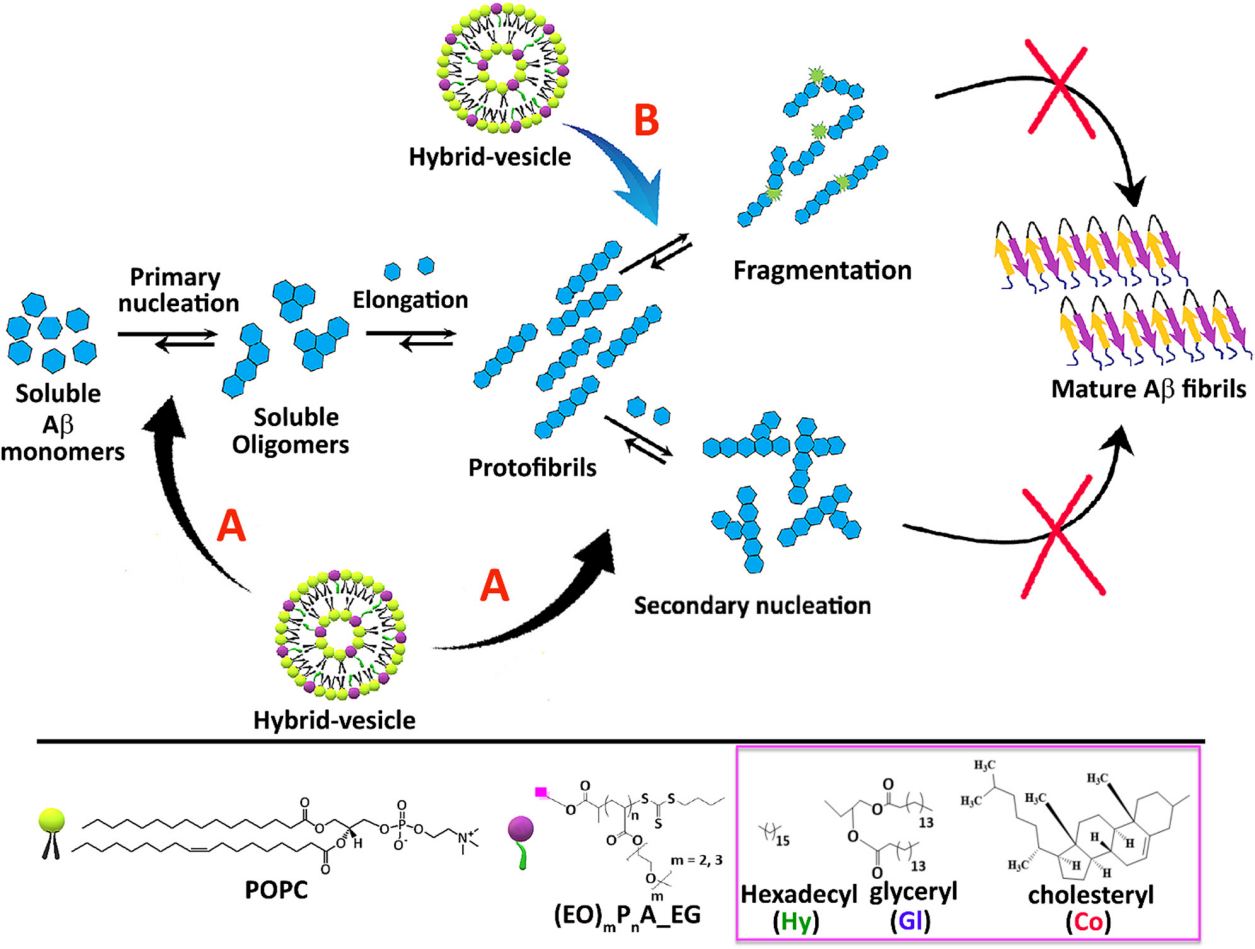
Lipid–polymer hybrid-vesicles and their influence on Aβ_1–40_ fibrillation. We focus on primary and secondary nucleation, elongation, and fragmentation steps (A; primary, secondary nucleation and B; fragmentation microscopic processes). These hybrid-vesicles can interfere with all steps of this Aβ_1–40_ fibrillation process, bearing the polymers, (EO)_*m*_P_*n*_A_**EG**, in variable molar ratios. Molecular structures of the lipid and polymers are displayed at the bottom of the figure.

Similar to the counterintuitive effects of lipid bilayer physico–chemical properties on amyloid fibrillation, PEGylated lipids are interesting candidates representing pharmaceutically accepted drugs, with the ability to balance lipid hydrophobicity by PEG hydrophilicity.^39^ Therefore, they are potential candidates to inhibit amyloid-aggregation as determined in our previous work, displaying small, but significant effects^40^ with enhancements of the lag-times of Aβ_1–40/42_ peptide fibrillation by a factor of ∼12. We here investigate a set of potential inhibitors for Aβ_1–40/42_ peptide fibrillation by a novel approach based on hybrid-vesicles, composed of model peptide (POPC) hybridized with hydrophilic polymers (hydrophobic and membrane-anchored, see Fig. 1) bearing PEO-sidechains. In the current investigations we delved into the impact of fine-tuning the hydrophilic–hydrophobic profile of the polymers on hybridization and amyloid formation akin to the pegylated lipids. Biophysical approaches were employed to gain insights into how the hybrid system modulates the fibrillation and conformational changes, particularly upon binding with the hybrid-vesicles. Via *in vitro* approaches, we elucidated the inhibition potential of those vesicles, together with analyzing of early stages of fibrillation and the associated nucleation events.

## Result and discussion

Conceptually we have used vesicles, composed of POPC as the main lipid, bearing an added hydrophilic polymer, safely embedded into the vesicle *via* a lipid anchor (Fig. 1). Tuning of the polymer's hydrophilicity is accomplished by changing the length of the ethylene-units at the monomers from either two (DGME) or three (TGME) ethylene oxide (EO) units, with the overall polymer's molar mass ranging from 2800 to 9950 Da. Lipid anchors (hexadecyl, glyceryl and cholesteryl groups) were used to stably incorporate those hydrophilic polymers into the POPC vesicles, subsequently probing their influence on Aβ_1–40_ fibrillation as outlined in Fig. 1. Polymers were prepared by RAFT (Reversible Addition Fragmentation Chain Transfer) polymerization, finely tuning their hydrophilic–hydrophobic profile by controlling parameters such as the degree of polymerization (*n*), the number of EO (ethylene oxide) units in the polymer backbone, and the incorporation of anchoring groups (such as membrane lipids and hydrophobic moieties).^40^ The synthesis and characterization of the so tuned polymers is described in the ESI‡ (see Scheme S1 and Fig. S1–S2) together with structural details of the selected polymers presented in Table 1. Biophysical approaches such as thioflavin T (ThT) fluorescence kinetic study, CD-spectroscopy, and TEM were employed to quantitatively and qualitatively explicate the impact of hybrid-vesicles on Aβ_1–40_ fibrillation. Moreover, the mechanistic insights of how these hybrid-vesicles influence Aβ_1–40_ aggregation were further investigated using the established Nature Protocol adopted in Amylofit.^41^

**Table 1:** Composition of hybrid-vesicles and the embedded polymers bearing the anchoring moieties (Hy, Gl, Co)

Entry	Core lipid conc. (mM)	Embedded polymer	Anchored group	*M*_n_ a (gmol^−1^)	Polymer incorporation (mol%)
1	POPC (1.5 mM)	(EO)_2_P_19_A_Hy	Hexadecyl (Hy)	3800	(5–20)%
2		(EO)_2_P_39_A_Hy		7250	
3		(EO)_3_P_12_A_Hy		3100	
4		(EO)_3_P_26_A_Hy		6150	
5		(EO)_2_P_22_A_Gl	Glyceryl (Gl)	4600	(5–20)%
6		(EO)_2_P_44_A_Gl		8450	
7		(EO)_3_P_11_A_Gl		3200	
8		(EO)_3_P_42_A_Gl		9950	
9		(EO)_2_P_23_A_Co	Cholesteryl (Co)	4600	(5–20)%
10		(EO)_2_P_48_A_Co		9000	
11		(EO)_3_P_10_A_Co		2800	
12		(EO)_3_P_18_A_Co		4550	
13		(EO)_3_P_52_A_Co		8000	

a Molar mass (*M*_n_) of the polymers confirmed *via*^1^H-NMR in CDCl_3_.

### Formation of hybrid-vesicles

Hybrid-vesicles were obtained by mixing POPC (as a core lipid component) with the respective polymers, carefully tuned by embedding anchoring groups, modulating their chain length (molecular weight), and add a specific number of EO units onto the polymer backbone. These so-tuned polymer characteristics allow embedding into the POPC-vesicles and further tune their outside-surface properties, resulting in hybrid-vesicles. This allowed a variable amount (5 to 20 mol%) of polymers in the hybrid-vesicles, modulating the hydrophilicity of the outer surface of these hybrid-vesicles.

**Fig. 2:**
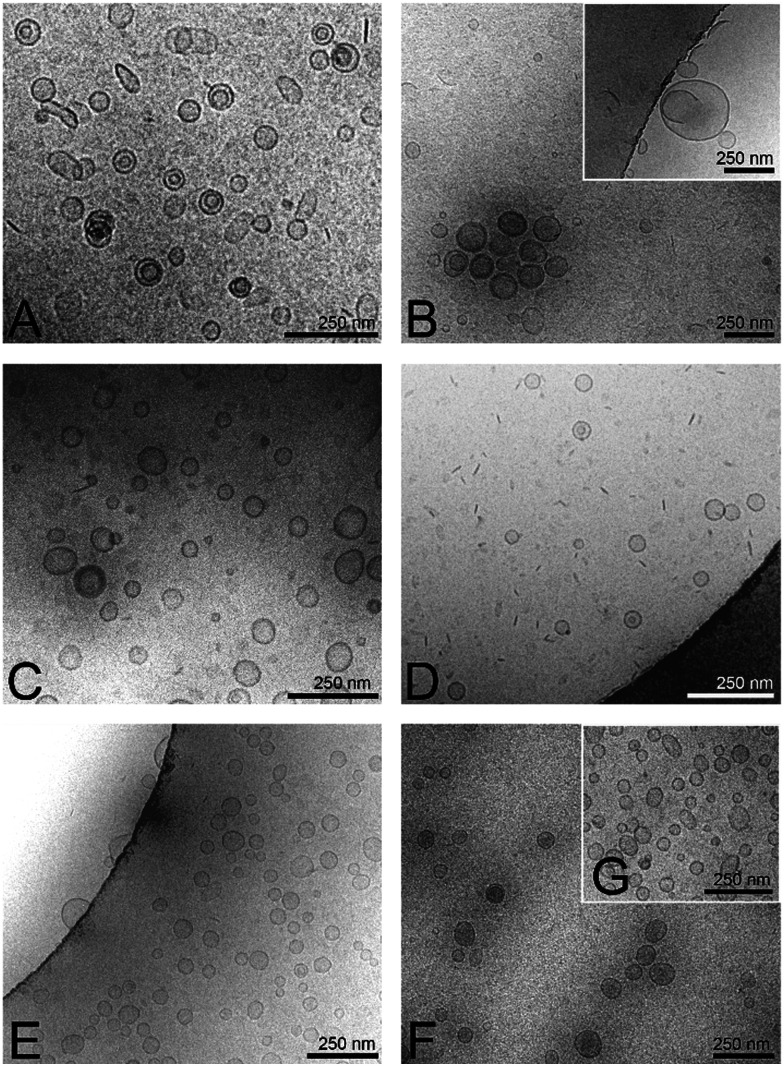
Cryo-TEM images of hybrid-vesicles with a scale bar of 250 nm. (A) (EO)_3_P_26_A_Hy_20 mol%, (B) (EO)_3_P_11_A_Gl_5%, (C) (EO)_3_P_42_A_Gl_5%, (D) (EO)_3_P_42_A_Gl_20%, (E) (EO)_2_P_48_A_Co_10%, (F) (EO)_3_P_18_A_Co_10% and (G) (EO)_3_P_52_A_Co_10% polymers embedded in POPC lipid.

Incorporating polymers with variable properties and amounts into the hybrid-vesicles posed a challenge as this alters the physio–chemical properties of the resulting hybrid-vesicles. We employed a modified extrusion process for the hybridization of POPC vesicles to generate hybrid-vesicles within a desirable size range of 55–200 nm (see Table S1, ESI‡). Lipids and polymers were mixed in water-free chloroform/methanol (2/1) at room temperature, followed by film formation and solvent removal. Subsequently, hybrid-vesicles were prepared in a 50 mM Na_2_HPO_4_ buffer solution supplemented with 150 mM NaCl at pH 7. 4 and a modified extrusion process. The buffer solution of the lipid–polymer mixture was extruded through a 400 nm polycarbonate (PC) membrane, followed by 200 nm and 100 nm PC membranes to obtain the desired size range of the hybrid-vesicles (details in ESI‡).^42^ The maxima of the narrow size distribution curves of dynamic light scattering (DLS) displayed the desired size range, while *cryo* transmission electron microscopy (Cryo-TEM) images shown in Fig. 2 and Fig. S4 (ESI‡) further confirmed the formation of hybrid-vesicles. The hybrid-vesicles exhibited stability at 4 °C for several hours, providing a sufficiently good timeframe for utilizing them in subsequent Aβ_1–40_ fibrillation kinetic investigations.

To ensure the complete embedding of the polymers into the hybrid-vesicles and quantify the amount of polymers incorporated inside the hybrid-vesicles, ^1^H- and ^31^P-NMR of the hybrid-vesicles bearing 5–15 mol% of (EO)_2_P_48_A_**Co** were performed and compared to the pure POPC vesicles (presented in Fig. S3, ESI‡). Proton NMR of the hybrid-vesicles confirmed the incorporation of the polymers into the hybrid-vesicles and enabled quantification of their amounts (see Fig. S3, ESI‡) by integration of resonances of the polymer *vs.* the lipid. The lipid head groups and their surrounding environment, especially their polarity are also sensitive to ^31^P-NMR spectroscopy; therefore, the chemical shift of ^31^P-NMR clearly indicates the incorporation of polymers in hybrid-vesicles.^43–45^ Consequently, the chemical shift for pure POPC vesicles shifted from approximately −0.6 ppm to 0.3 ppm upon incorporation of the polymers inside the POPC-vesicles in amounts of up to 15%. This change in the chemical shift serves as a clear indicator of successful polymer incorporation, with the amphiphilic environment introduced by the polymers likely contributing to this effect. Moreover, confocal fluorescence microscopy of Rh-DPPE (Lissamine rhodamine B-1,2-dipalmitoyl-*sn*-glycero-3-phosphoethanolamine) fluorescently dye-labeled giant unilamellar vesicles (GUVs) composed of pure POPC and hybrid GUVs containing 5 mol% of (EO)_2_P_48_A_**Co** revealed no unusual membrane inhomogeneities in the POPC membrane caused by the polymer's incorporation (Fig. S20, ESI‡).^46^ The physical integrity of the hybrid-GUVs played a crucial role in the functionality, together with the proven stability of the polymers when embedded inside the membrane (as checked by MALDI-ToF measurements, see Fig. S22 and S23, ESI‡). This was further corroborated by monitoring the zeta potential (Fig. S21C, ESI‡) and size measurements (Fig. S21A, ESI‡) of the hybrid-vesicles over time and temperature (Fig. S21B, ESI‡).

### Modulating fibrillation with hybrid-vesicles

**Fig. 3:**
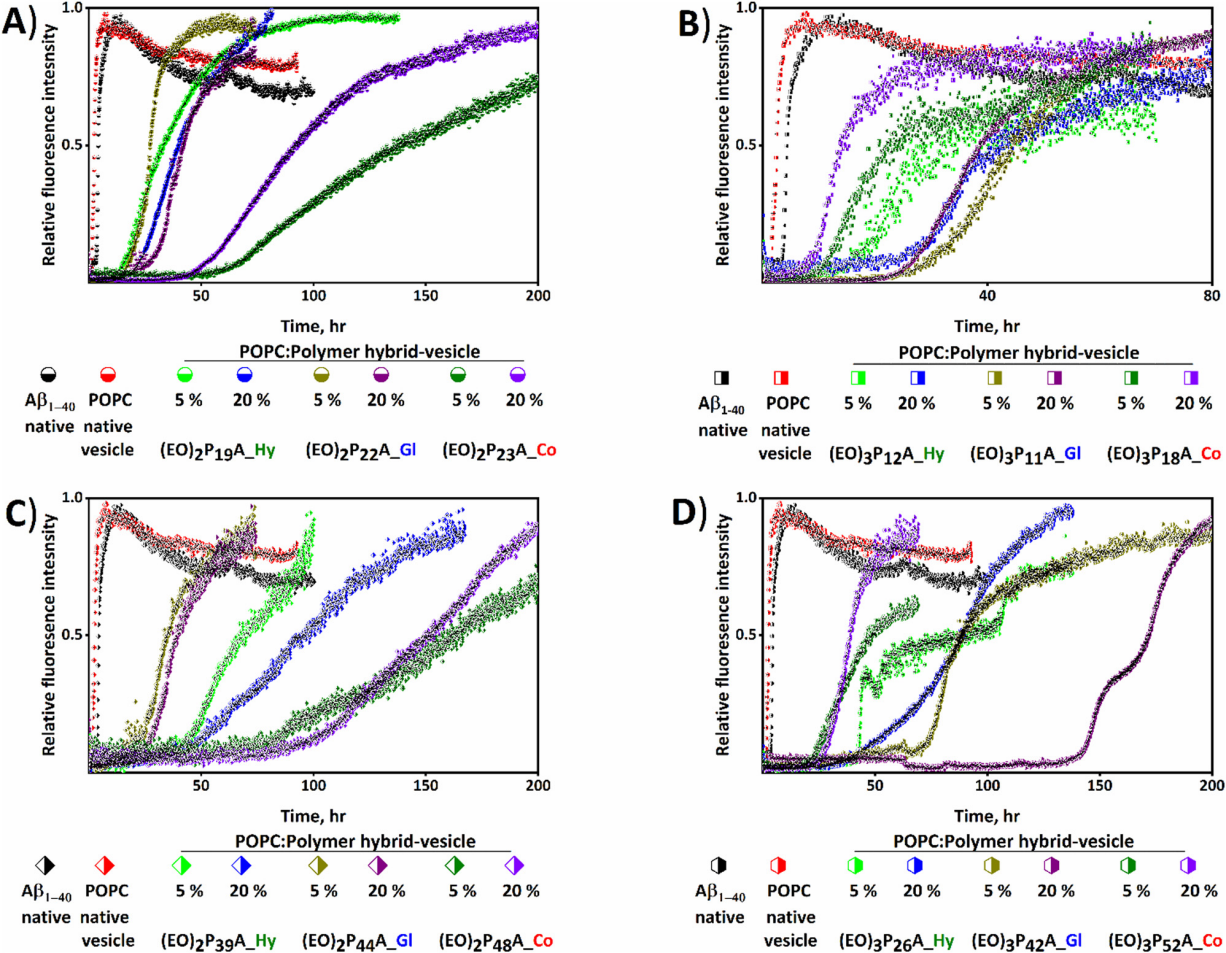
Analysis of the effects of hybrid-vesicles on the aggregation profile of Aβ_1–40_. Thioflavin T (ThT) monitored kinetic profiles for the aggregation of Aβ_1–40_ in phosphate buffer solutions of pH 7.4 in the presence of POPC and hybrid-vesicles at 37 °C are shown in (A) to (D). The black error bars represent the standard deviation of three normalized independent time-resolved ThT fluorescence. Fibrillation kinetics of native Aβ_1–40_ (black diamond shape) and in the presence of POPC- and hybrid-vesicles embedded with comparable low (A) and (B) and high (C) and (D) molecular weight polymers bearing two and three ethylene oxide (EO) units, respectively. The polymers and their respective molar fraction in the hybrid-vesicles are presented in each panel with distinctive colors. Hy, Gl, and Co represent the hexadecyl, glyceryl, and cholesteryl end groups of the embedded polymers.

**Fig. 4:**
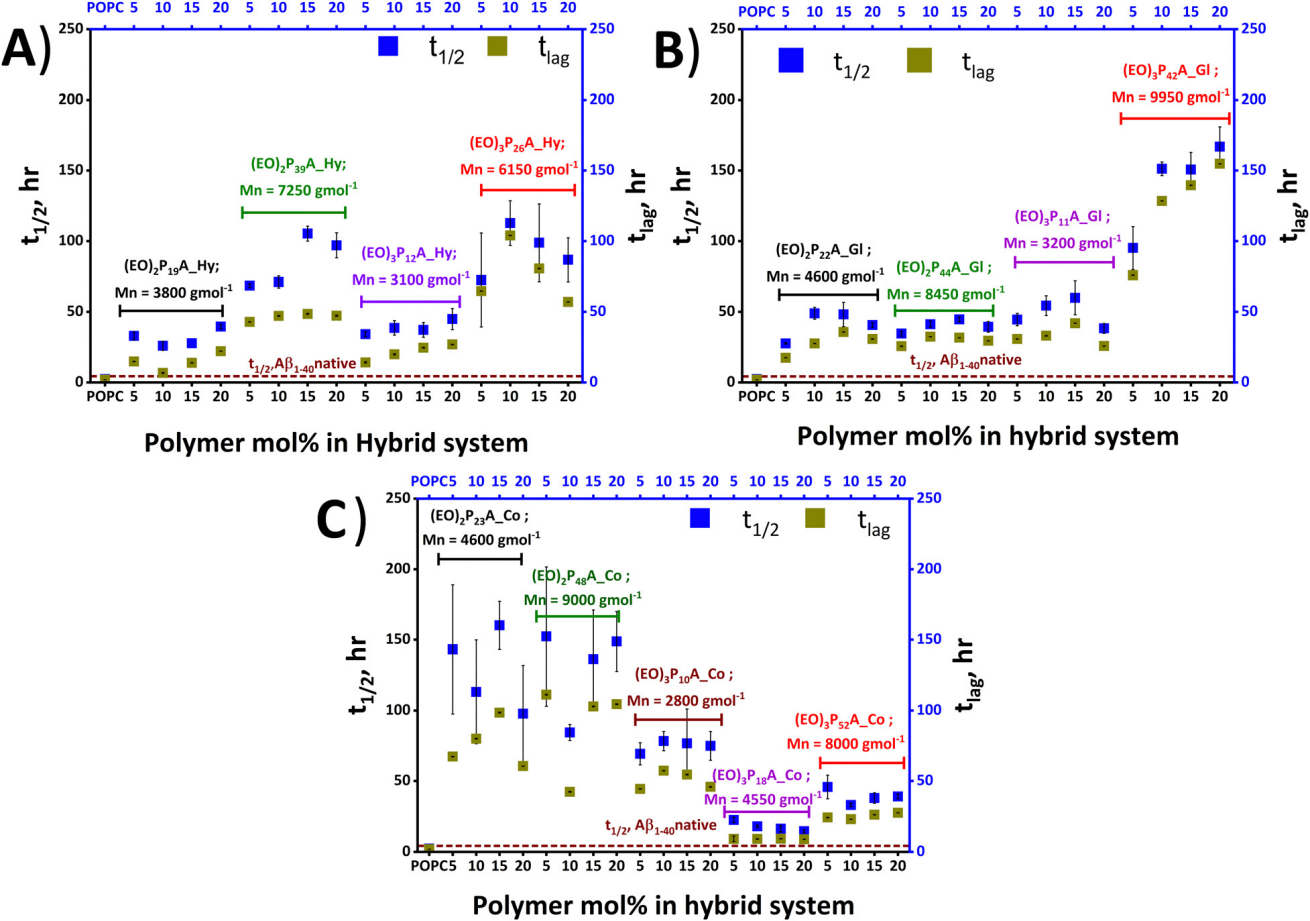
Quantitative determination of the impact of hybrid-vesicles on Aβ_1–40_ aggregation. The lag time, *t*_lag_, and half time, *t*_1/2_ are shown in each panel to represent the *t*_lag_ and *t*_1/2_ estimated from the fitting of three individual Aβ_1–40_ fibrillation kinetics in the presence of POPC and POPC–polymer vesicles. *t*_lag_ and *t*_1/2_ are plotted against the concentration of the incorporated polymers inside the hybrid-vesicles and compared with the *t*_1/2_ of Aβ_1–40_ in the absence of any vesicles in the solution. Impact of (A) hexadecyl; Hy, (B) glyceryl group; Gl and (C) cholesteryl group; Co anchored polymers in hybrid-vesicles on Aβ_1–40_ fibrillation.

We studied the influence of the hybrid-vesicles on Aβ_1–40_ fibrillation, focussing on macroscopic changes (determining lag time; *t*_lag_ and half time; *t*_1/2_ of fibrillation by ThT assays) and morphological characteristics of the resulting aggregates *via* TEM. For the ThT assays purified Aβ_1–40_ peptide was dissolved and sonicated in buffer, the same buffer used for hybrid-vesicles preparation, to achieve a nucleation-free monomeric Aβ_1–40_ for the subsequent ThT assay.^47^ The freshly prepared POPC and hybrid-vesicles were added to the monomeric fibrillating amyloid-Aβ_1–40_ proteins, using a ratio of [lipid/peptide] = [150/1], shortly before the ThT-assays (details of the fibrillation kinetics in ESI‡).^42^ Utilizing *in vitro* biophysical assays by ThT fluorescence allowed tracking of the fibrillation kinetics and provided a deeper insight into both, quantitative parameters (from *t*_lag_ and *t*_1/2_) and further qualitative aspects (including the mechanism of interactions). In all cases, the hybrid-vesicles, containing the embedded polymers, interfered significantly with the fibrillation processes and resulted in elongated fibrillation times, proving that all investigated hybrid-vesicles exerted a pronounced influence on Aβ_1–40_ fibrillation, as evidenced by the graphical representation of Aβ_1–40_ fibrillation kinetics shown in Fig. 3 and Fig. S5 (ESI‡). It was noted that fibrillation of Aβ_1–40_ had accelerated slightly (*t*_1/2_ ≈ 2.5 hours and *t*_lag_ ≈ 2 hours) in the presence of native POPC vesicles devoid of polymers compared with the native Aβ_1–40_ (*t*_1/2_ ≈ 4.5 hours and *t*_lag_ ≈ 4 hours). The fibrillation kinetics of both native Aβ_1–40_ and polymer-free POPC vesicles were considered as a reference (see Fig. 3, 4 and Fig. S5, ESI‡). Subsequently, the *t*_lag_ and *t*_1/2_ obtained from the fitting of kinetics are summarized in Fig. 4 and Table S1 (ESI‡).

We firstly focussed on a set of POPC; polymer hybrid-vesicles bearing 5 mol% polymers to study the impact of polymer properties like hydrophilicity (number of EO units, degree of polymerization; *n*) and the hydrophobicity (nature of the anchoring groups; hexadecyl (Hy), glyceryl (Gl) and cholesteryl (Co)) on amyloid fibrillation. Thus, the nature of the tethering groups exhibited a profound impact on Aβ_1–40_ fibrillation, in all cases inducing a significant elongation of the fibrillation. In the presence of the hexadecyl group bearing polymer, (EO)_2_P_19_A_**Hy**; 3800 Da inhibition of fibrillation was observed with a *t*_1/2_ of ≈ 40 hours and *t*_lag_ ≈ 15 hours, whilst the (EO)_2_P_22_A_**Gl**; 4600 Da polymer with a glyceryl group exhibited lower retardation effects (*t*_lag_ ≈ 18 hours and *t*_1/2_ ≈ 28 hours) of fibrillation. The strongest retardation was observed when the cholesteryl group anchored (EO)_2_P_23_A_**Co**; 4600 Da polymer, incorporated in the POPC-hybrid-vesicles with *t*_1/2_ ≈ 143 hours and *t*_lag_ ≈ 67 hours (Fig. 3A, 4 and Table S1, ESI‡).

There also was a strong influence of the length of the side-chain-EO-groups (2, 3) on fibrillation, when comparing similar polymers of otherwise similar structure. Among the previously mentioned polymers, the (EO)_2_P_22_A_**Gl** polymer exhibited a shorter retardation time compared to the other two mentioned counterparts. However, when the number of EO units increased from 2 to 3 a significant fibrillation inhibition was attained in (EO)_3_P_11_A_**Gl**, which was quantified with a *t*_1/2_ of 28 hours to 47 hours and *t*_lag_ of 18 hours to 31 hours (see Fig. 3A, B, 4 and Table S1, ESI‡). We further studied the influence of chain length of the polymer upon the fibrillation of Aβ_1–40_.^40^ By comparing (EO)_3_P_42_A_**Gl** with increasing molar mass from 3200 Da to 9950 Da but otherwise identical number of EO units and the anchoring group, a substantial elongation of the lag time ≈76 hours for 5 mol% (EO)_3_P_42_A_**Gl** polymer bearing hybrid-vesicles compared with the native Aβ_1–40_ lag time (≈4 hours) was observed (details in Fig. 3C, 4 and Table S1, ESI‡).

Other than the above-mentioned inherent properties of polymers, the amount of incorporated polymers into hybrid-vesicles was also crucially important for altering the physio–chemical properties of the vesicles and concomitantly the fibrillation behaviour. In every tested lipid–polymer composition, 5 to 20 mol% polymers within POPC lipid, the inhibition of fibrillation persisted. However, no clear trend emerged correlating an increase in polymer content with greater fibrillation retardation. Focusing on a single set of hybrid-vesicles containing (EO)_3_P_10_A_**Co**, a *t*_lag_ ≈ 45 hours was observed when the hybrid-vesicles were bearing only 5 mol%, changing only slightly the *t*_lag_ (≈46 hours) when using 20 mol%. A more pronounced shift in the aggregation *t*_1/2_, from ≈69 hours to ≈75 hours was found between these two concentrations. Further, quantitative analysis (*t*_lag_, and *t*_1/2_) of the fibrillation kinetics are displayed in Fig. 4, Fig. S6 and Table S1 (ESI‡).

Besides the quantitate estimation of *t*_lag_ and *t*_1/2_ from the fibrillation kinetics, the total amount of fibrillar aggregates can also be calculated from the raw ThT intensities upon reaching their maximum (see Fig. S7 and S8, ESI‡). The collective amount of fibrils was reduced substantially with the quantity of mature fibrils further reduced in the presence of all hybrid-vesicles regardless of their embedded polymer amount and properties (see Fig. S9, ESI‡).

### Fibrillation kinetics and mechanistic understanding of the hybrid-vesicles interaction

**Fig. 5:**
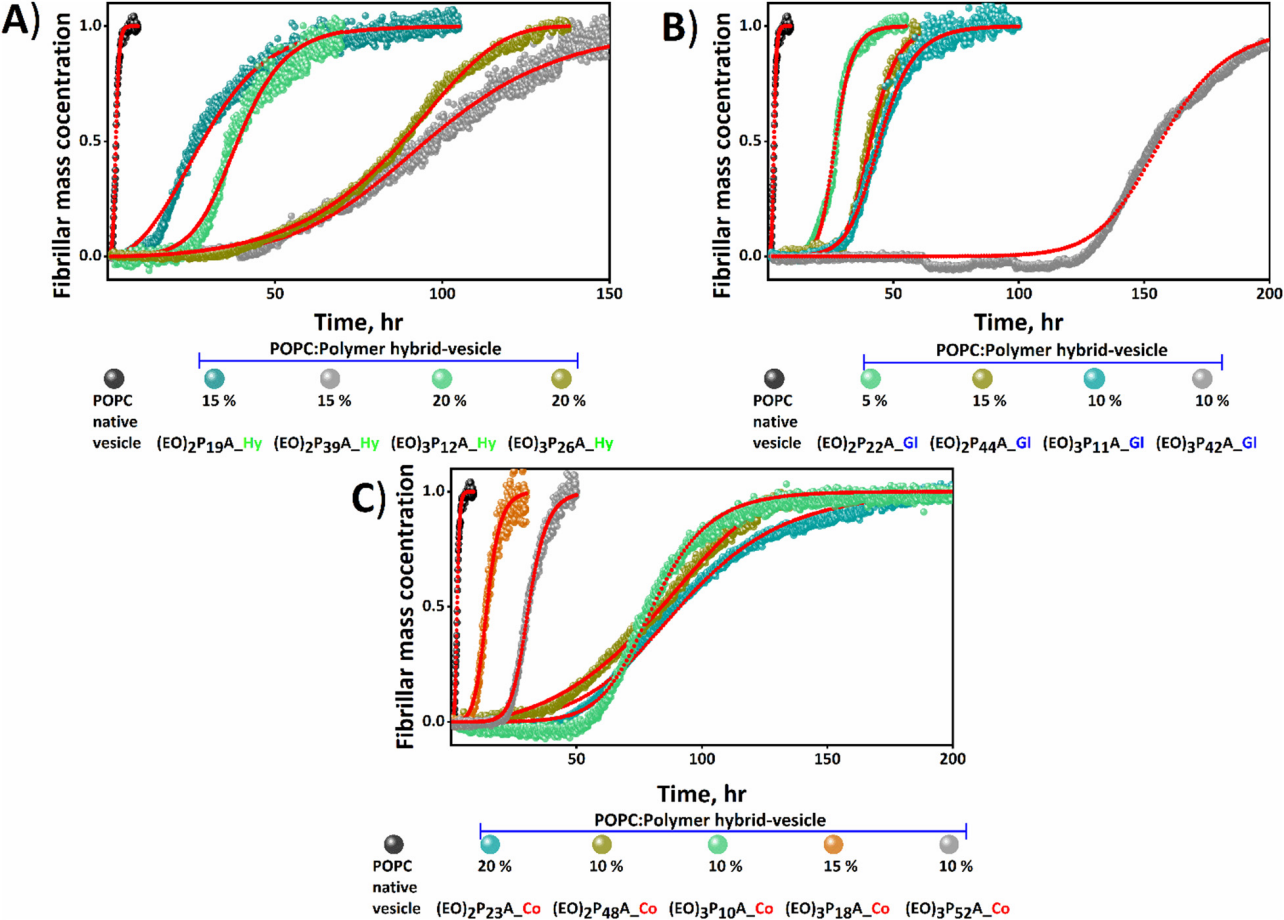
Correlation between nature of the hybrid-vesicles and Aβ_1–40_ aggregation. Mean normalized kinetic reaction profiles fitted globally in Amylofit keeping the nucleus size (*n*_*c*_, primary = *n*_2_, secondary = 2)^30,51,53,54^ and initial monomer concentration (10 μM) of Aβ_1–40_ constant while fitting. The red dotted lines represent the fitting of the curve. Most hybrid-vesicles (presented in A, B and C panels) interfered the primary (1°) and secondary (2°) nucleation pathways of Aβ_1–40_ aggregation, whereas the hybrid-vesicles bearing (EO)_3_P_26_A_**Hy**_20% (panel A) and (EO)_2_P_48_A_**Co**_10% (panel C) polymers interfered the elongation pathway along with the nucleation pathways. Both POPC and hybrid-vesicles including polymers embedded are presented at the bottom of each panel with distinctive colours of the circle.

**Fig. 6:**
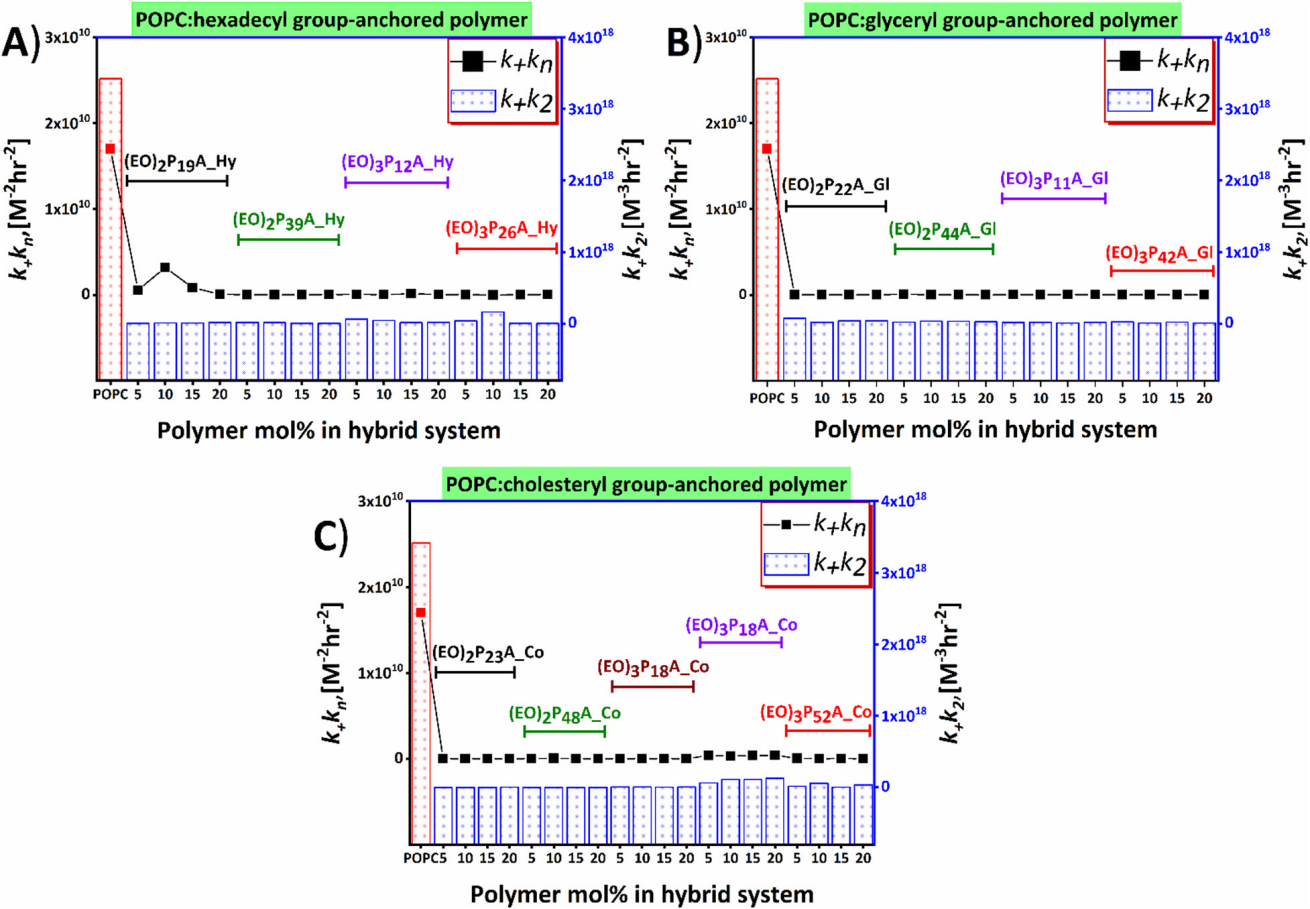
Influences of lipid–polymer hybrid-vesicles on primary and secondary nucleation process of Aβ_1–40_ aggregation as analysed by Amylofit. Amount of polymers embedded in hybrids plotted against the *k*_+_*k*_*n*_ and *k*_+_*k*_2_ for variable end groups hexadecyl (Hy), glyceryl (Gl), and cholesteryl (Co) bearing polymers are presented in panels (A), (B), and (C), respectively. Polymers are marked with specific colors. The *k*_+_*k*_*n*_ (for primary nucleation) and *k*_+_*k*_2_ (for secondary nucleation) of Aβ_1–40_ in the presence of hybrid-vesicles are compared with the POPC vesicles and obtained from the global fitting of ThT fluorescence kinetics.

Time-resolved ThT fluorescence kinetics can unveil microscopic processes such as nucleation (primary and secondary), fragmentation, elongation, and mature fibril formation during the transformation of the functional soluble state of the proteins to insoluble pathogenic solid aggregates. Mathematical models incorporating these microscopic processes offer a numerical survey of the fibrillation kinetics, including both seeded (involving small fibrils or pre-fibrillar aggregates) and unseeded (dependent on monomers) models, all contributing to the mechanistic understanding of the chemical progression to amyloids over time quantitatively.^48–52^ We here have used the open-access online fitting platform Amylofit^41^ to decipher the mechanism underlying amyloid aggregation and how the added hybrid-vesicles could interfere with fibrillation as observed in the ThT fluorescence kinetics. Fitting of the experimental ThT kinetics allows extraction of the integrated rate laws of the specific microscopic steps. Comparison of the rate laws enables interpretation of the interference by the materials semi-quantitatively. Our experimental kinetic data were fitted with the microscopic step-variable kinetic models to identify the most suitable model. Testing several models, the global analysis of the ThT fluorescence traces obtained for most hybrid-vesicles fitted reasonably with the unseeded version of the secondary nucleation dominated model. However, for some hybrid-vesicles bearing mainly the cholesteryl-anchored polymers, the unseeded version of fragmentation and secondary nucleation dominated model globally fitted suitably (Fig. 5 and Fig. S6, ESI‡). A brief summary of the hybrid-vesicles influence on Aβ_1–40_ fibrillation is presented in Table 2. All corresponding integrated rate laws from a quantitative analysis using Amylofit are listed in Table S2 (ESI‡) and graphically represented in Fig. 6. The combined rate constants *k*_+_*k*_*n*_ (primary nucleation rate) and *k*_+_*k*_2_ (secondary nucleation rate) of Aβ_1–40_ fibrillation in the presence of hybrid-vesicles allow a rough correlation of inhibition of fibrillation in microscopic processes. POPC-vesicles only marginally accelerated the fibrillation in comparison to native Aβ_1–40_, while all hybrid-vesicles delayed fibrillation significantly. Subsequently, a substantial perturbation of both primary (*k*_+_*k*_*n*_) and secondary (*k*_+_*k*_2_) nucleation rates of Aβ_1–40_ in the presence of POPC–polymer vesicles compared to POPC vesicles was observed (see Fig. 6 and Table S2, ESI‡). Additionally, some of the hybrid-vesicles, particularly those embedding cholesteryl-anchored polymers, interfered with the fragmentation and elongation steps compared to the other investigated hybrid-vesicles as reflected in the combined rate laws (*k*_−_*k*_+_) listed in Table S2 (ESI‡). The extended fibrillation, as indicated by their microscopic rate constants, was affected by the lipid's hydrophobicity or proposedly by steric hindrance arising from non-productive interactions with the hydrophilic portion of the polymer to the hybrid-vesicles surface.^28,39^ These interactions could potentially alter the microscopic processes of fibrillation, including the morphological transition of the aggregates.

**Table 2:** Summary of microscopic processes of Aβ_1–40_ fibrillation influenced by the hybrid-vesicles as evaluated by Amylofit

Polymer	Polymer mole fraction (%) incorporated in POPC: Polymer hybrid system	Mechanism of Aβ_1–40_ aggregation interference *via* hybrid-vesicles
(EO)_2_P_19_A_Hy	(5–20) %	Primary and secondary nucleation of Aβ_1–40_ aggregation interfered *via* hybrid-vesicles
(EO)_2_P_39_A_Hy	(5–20) %	
(EO)_3_P_12_A_Hy	(5–20) %	
(EO)_3_P_26_A_Hy	5%, 10%	
(EO)_2_P_22_A_Gl	(5–20) %	
(EO)_2_P_44_A_Gl	(5–20) %	
(EO)_3_P_11_A_Gl	(5–20) %	
(EO)_3_P_42_A_Gl	(5–20) %	
(EO)_2_P_23_A_Co	(5–20) %	
(EO)_3_P_10_A_Co	5%, 10%, 20%	
(EO)_3_P_18_A_Co	(5–20) %	
(EO)_3_P_52_A_Co	5%, 10%, 20%	
(EO)_3_P_26_A_Hy	15%, 20%	Primary, fragmentation and secondary nucleation of Aβ_1–40_ aggregation interfered *via* hybrid-vesicles
(EO)_3_P_10_A_Co	15%	
(EO)_2_P_48_A_Co	(5–20) %	
(EO)_3_P_52_A_Co	15%	

### Morphological transitions of aggregates upon interaction with hybrid-vesicles

**Fig. 7:**
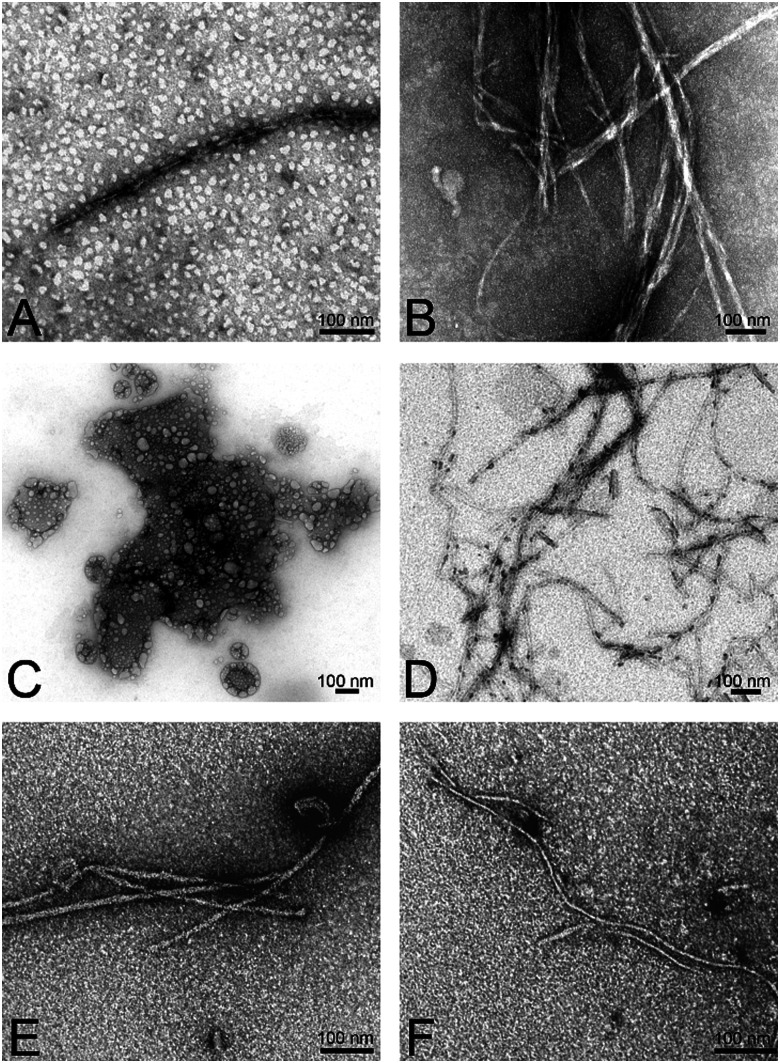
Morphological analysis hybrid-vesicles interactions with Aβ_1–40_ fibrils. Aβ_1–40_ fibrils formed in ThT assays in the presence of hybrid-vesicles containing (A) (EO)_3_P_12_A_Hy_10%, (B) (EO)_3_P_26_A_Hy_5%, (C) (EO)_2_P_44_A_Gl_20%, (D) (EO)_2_P_44_A_Gl_20%, (E) (EO)_2_P_23_A_Co_20% and (F) (EO)_3_P_10_A_Co_10% polymers. A negative stain of uranyl acetate was used to record TEM images. The scale bar of the images represents 100 nm.

The reduced ThT fluorescence was a clear indication of structural changes in Aβ_1–40_ aggregates upon interaction with the hybrid-vesicles by changing into the aggregated β-sheets structures. To track eventual morphological changes in the Aβ_1–40_ aggregates induced by the POPC and hybrid-vesicles, circular dichroism (CD) spectroscopy and transmission electron microscopy (TEM) imaging experiments were performed on the fibrillar aggregates. The images presented in Fig. 7 and Fig. S15 (ESI‡) revealed the presence of secondary structures of Aβ_1–40_ aggregates after interactions with the vesicles. The incubation of monomeric Aβ_1–40_ with hybrid-vesicles is accompanied by a structural shift from enriched β-sheets to random coil conformations. This shift was distinct from the structural characteristics observed for native Aβ_1–40_ (as evidenced by CD-spectra presented in Fig. S12–S14, ESI‡). Furthermore, the CD-spectra of the aggregates validated the findings of fibril load, through structural elucidation *via* the BeStSel (Beta Structure Selection) algorithm, an online platform to determine secondary structures of proteins,^55^ unveiled the presence of α-helix and irregular structures along with compact β-sheets in the aggregates (see summary in Table S3, ESI‡). Subsequently, TEM images were used to crosscheck the structural transitions of the Aβ_1–40_ aggregates, revealing the presence of short, less compact aggregates to dense long entwined fibrils or no apparent fibrillar structures. Hybrid-vesicles with respective polymers and compositions are presented according to the microscopic steps of the Aβ_1–40_ aggregation interrupted upon interactions with the hybrid-vesicles.

## Conclusion

As neuronal membrane components and surfaces play a crucial role in nucleating the pathogenicity of the Aβ_1–40_,^14,32,33,56,57^ we here studied the impact of artificial hybrid-vesicles on amyloid fibrillation. Amphiphilic polymers were embedded into hybrid-POPC vesicles and were probed as an *in vitro* strategy to modulate Aβ_1–40_ fibrillation. Upon incubation of non-aggregated Aβ_1–40_ with hybrid-vesicles, a significant inhibition of fibrillation by an increase in the fibrillation times (both *t*_lag_ and *t*_1/2_) was observed for all hybrid-vesicles bearing the embedded amphiphilic polymers with factors of up to 30-fold, compared to the native vesicles devoid of the incorporated polymer. Compared to our previous investigations with the same polymers being non-embedded inside the liposomes,^40^ we here observed a significant elongation of the fibrillation times of a factor 1.5–2, which hints at a contribution of the liposomal surfaces. A systematic mechanistic analysis by the program Amylofit allowed to track kinetic parameters (*k*_+_*k*_*n*_ and *k*_+_*k*_2_) and thus primordial states of the Aβ_1–40_ aggregation. The polymer-modified liposomes interfere with amyloid nucleation processes, affecting both primary and secondary nucleation rate constants (*k*_+_*k*_*n*_ and *k*_+_*k*_2_). This suggests an interaction on the molecular level between the hybrid-vesicles and the proteins also visible by the reduced fibril loads and the morphological transition of the highly compact long unbranched fibrils to very short uncompacted fibrils or amorphous aggregates. This remarkable inhibitory behaviour of the hybrid-vesicles may be attributed to an umbrella effect over the hybrid-vesicles created by the tuned hydrophilic–hydrophobic profile of the embedded polymers.^58^ Polymer-induced shielding effects over the hybrid-vesicles surface could ultimately resist the interactions (both electrostatic and hydrophobic interactions) between the monomers and hybrids, thereby mitigating amyloid fibrillation.

It can also be considered that attenuated fibrillation is linked to the net charge of the POPC-hybrid-vesicles surface, altered by the amphiphilicity of the polymers that potentially facilitates repulsion to accumulate the negatively charged Aβ_1–40_ monomers.^59–63^ This overall stresses the importance of the outer decoration of vesicular, membrane-like surfaces in the fibrillation kinetics of the Aβ_1–40_ protein, further supported by CD and TEM. We think that our current work significantly extends the understanding of the role of tailored membrane physio–chemical properties in the context of their inhibitory role in Aβ_1–40_ pathogenesis *via in vitro* studies, stressing the influence of the early stages of Aβ_1–40_ aggregation and underscoring the impact of vesicular membrane in the process of amyloid disease progression.

## Author contributions

N. S. did the synthesis of polymers, hybrid-vesicles preparation, and fibrillation kinetics assays. S. K. performed cryo TEM and TEM. The research was designed and the data was analyzed by N. S. and W. H. B. The manuscript was written by N. S. and W. H. B.

## Data availability

Materials, experimental procedures, NMR and mass spectra, raw data have been included in the ESI.‡

## Conflicts of interest

There are no conflicts to declare.

## Supplementary Material

CB-OLF-D4CB00217B-s001
