# Supplementary material for: Lipid–polymer hybrid-vesicles interrupt nucleation of amyloid fibrillation
Source: RSC Chem Biol. 2024 Oct 24;5(12):1248–58. doi: 10.1039/d4cb00217b (PMC11575630; doi:10.1039/d4cb00217b)
Supplement: CB-005-D4CB00217B-s001 [file CB-005-D4CB00217B-s001.pdf]

Supplementary Information (SI) for RSC Chemical Biology.
This journal is © The Royal Society of Chemistry 2024

Supplementary Information

Lipid-Polymer Hybrid-Vesicles Interrupt Nucleation of Amyloid Fibrillation

Newton Sen[a], Stephanie Krüger[b] and Wolfgang H. Binder[a]*

[a] Macromolecular Chemistry, Institute of Chemistry, Faculty of Natural Science II (Chemistry, Physics and Mathematics)Martin Luther University Halle-Wittenberg, von-Danckelmann-Platz 4, D-06120 Halle, Germany. E-mail: wolfgang.binder@chemie.uni-halle.de

[b] Martin-Luther University Halle-Wittenberg, Biocenter, Weinbergweg 22, D-06120 Halle (Saale), Germany.

Materials

The purified 1-palmitoyl-2-oleoyl-glycero-3-phosphocholine (**POPC**) lipid powder and thioflavin T (ThT) were procured from Sigma Aldrich. The 400, 200 and 100 nm polycarbonate (**PC**) membranes for vesicles preparation were also purchased from Sigma Aldrich. Water-free HPLC grade methanol and chloroform solvents were bought from VWR Deutschland. Azobisisobutyronitrile (AIBN, 98%) initiator and Anhydrous Dimethylformamide (DMF) were purchased from Sigma Aldrich. The $(EO)_2P_{19}A$_**Hy**, $(EO)_2P_{39}A$_**Hy**, $(EO)_3P_{12}A$_**Hy**, $(EO)_3P_{26}A$_**Hy**, $(EO)_2P_{22}A$_**Gl**, $(EO)_2P_{44}A$_**Gl**, $(EO)_3P_{11}A$_**Gl**, $(EO)_3P_{42}A$_**Gl**, $(EO)_2P_{23}A$_**Co**, $(EO)_2P_{48}A$_**Co**, $(EO)_3P_{10}A$_**Co**, $(EO)_3P_{18}A$_**Co** and $(EO)_3P_{52}A$_**Co** polymers were synthesized via reversible-addition-fragmentation chain transfer (RAFT) polymerization method.[1] Fmoc based solid-phase peptide synthesize (SPPS) method was employed to synthesize $A\beta_{1\text{-}40}$ (DAEFRHDSGY EVHHQKLVFF AEDVGSNKGA IIGLMVGGVV) peptide at the peptide Core Unit of Leipzig University.

Methods

Synthesis of Polymers

The reversible addition-fragmentation chain transfer (RAFT) polymerization was used to synthesize the polymers. For the end groups (**Hy** = hexadecyl; **G**l = glyceryl, **Co** = cholesteryl) fidelity of the polymers, the RAFT (CTA) agents possessing the **Hy**, **Gl** and **Co** were used. The monomers for the polymerization with the specific number of ethylene oxide (EO) units were synthesized.[1] Both monomers and RAFT agents were synthesized separately before starting the polymerization reaction.[1] Subsequently, The RAFT agents were used to synthesize the tuned polymers with defined end groups using AIBN initiator at 70 °C. The media of the polymer synthesis was a very small amount (approximately 1 mL) of DMF (see **Scheme 1**). The details of the synthesis of monomers, RAFT agents and polymers were reported previously.[1]

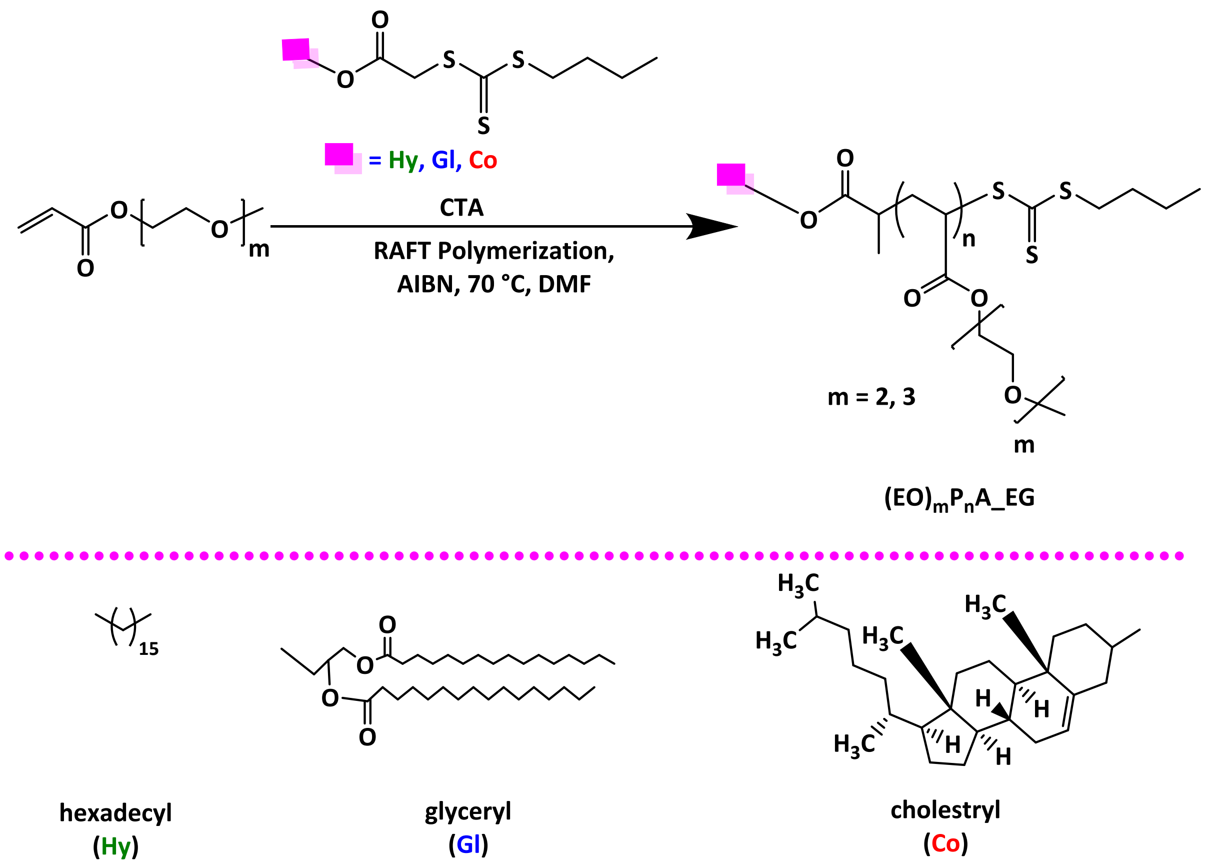

Scheme 1: Synthesis of $(EO)_mP_nA$_**EG** homopolymers with different end groups.[1]

To demonstrate the successful polymer synthesis, the solution polymerization of $(EO)_2P_{23}A$_**Co** is presented here. The polymerization was performed using the RAFT agent S-butyl-S-cholesteryltrithiocarbonate and AIBN initiator. The monomer was diethylene glycol methyl ether acrylate (**DEGA**). The molar ratio of monomer, RAFT agent and initiator was 22: 1: 0.1. All ingredients were mixed in 1.2 mL of dry DMF in a sealed vial with continuous stirring. Deoxygenation of the reaction mixture was performed by purging with Argon for 45 minutes. The reaction was continued for three hrs in 70°C pre-heated oil bath and quenched by opening the vial septum in a liquid nitrogen environment. The unreacted monomers and oligomers were eliminated by precipitating the polymers in cold n-hexane (excess amount). The remaining DMF was removed after drying the purified polymers in high vacuum for 2 weeks. The ^{1}H-NMR of DEGA monomer, S-butyl-S-cholesteryltrithiocarbonate and ESI-TOF Ms spectra of RAFT agent were presented in **Figure S16**, **S17** and **S18**, respectively.[1] As evidence of the successful synthesis of the polymers, the NMR and MALDI-tof of $(EO)_2P_{23}A$_**Co** polymer are shown here (see **Figure S1** and **S2**).

Characterization data of diethylene glycol methyl ether acrylate (DEGA):

^{1}H-NMR (500 MHz, $CDCl_3$): δ (ppm): 6.4 (ddd, C**H₂**=CH, $^1H_1$), 6.13 (m, C**H₂**=CH, $^1H_1$), 5.82 (dt, CH_2=C**H,** $^1H_2$), 4.31 (m, -OC**H₂**-, $^2H_4$), 3.73 (m, -OCH_2-C**H₂**-O-, $^2H_5$), 3.65 (m, -CH_2-O- C**H₂**-, $^2H_6$), 3.54 (m, - CH_2- C**H₂**-O, $^2H_7$), 3.37 (d, -O- C**H₃**, $^2H_8$) (**Figure S16**).

Characterization data of S-butyl-S-cholesteryltrithiocarbonate:

^{1}H-NMR (500 MHz, $CDCl_3$): δ (ppm): 5.40 – 5.34 (m, C**H**=CH, $^1H_{17}$), 4.77 (q, SC**H**CH_3, $^1H_6$), 4.69 – 4.58 (m, C**H**-O,$^1H_{10}$), 3.36 (td, SC**H₂**, $^2H_4$), 2.39 – 2.26 (m, $^2H_{12}$), 2.07 – 0.72 (m, $^{48}H_{1-3}$, 7, 9, 11, 15, 16, 18-20, 22-33, 35), 0.68 (s, C**H₃**, $^3H_{34}$) (**Figure S17**).

ESI-TOF MS: $[M+Na]^+$ = 629.34 (**Figure S18**).

Hybrid-vesicles preparation

A mixture of chloroform and methanol (2:1) solvent was used to dissolve both the POPC and polymers. The solutions of lipids and polymers were used further as stock solutions for hybrid-vesicles preparation. Varying molar concentrations (5-20 mol%) of polymers were mixed with 1.5 mM DOPC lipid solution. Lipid-polymer mixture was vortexed for 10 minutes to ensure uniformity of the mixture and subsequently, the solvent of the clear mixture was evaporated at minimum pressure using rotaevaporator to remove the organic solvent completely. After drying, a thin film of lipid and lipid-polymer was deposited on the glass vial wall. A white suspension was obtained after rehydration of dried film using 150 mM NaCl supplemented 50 mM Na_2HPO_4 buffer (pH 7.4) by vortexing for 5-10 minutes. Afterwards, the suspension was undergone freeze-thaw cycle (10 times). A modified extrusion method was employed to get hybrid-vesicles from the suspension.[2-4] First, the suspension was extruded twenty one (21) times through the 400 nm PC membrane, and subsequently through the 200 nm and 100 nm to obtain hybrid-vesicles. On the contrary, the POPC vesicles were obtained by extruding through the 400 and 100 nm PC membranes. All extrusion was performed via the mini extruder from Avanti polar Lipids at room temperature. Dynamic light scattering (DLS) was used to determine the size of the vesicles. The hybrid-vesicles used to investigate their influence on AB fibrillation kinetic assays, until then the vesicles were stored at 4°C.

Hybrid-vesicles size determination

DLS (Anton Paar Litsizer 500) was used to determine the size of the hybrid-vesicles at 25 °C. After the vesicle preparation, the vesicle solution was placed in the 70 μL disposable micro cuvettes. The vesicles diffusion co-efficient was used to determine the DOPC and hybrid-vesicles size. Diffusion coefficient was inferred using the autocorrelation function's scattered intensity. The Stokes-Einstein equation uses the diffusion co-efficient to deduce the size of the vesicles. The vesicles hydrodynamic diameter was calculated using integrated Anton Paar Kalliope Ver. 2.16.0 software.

Imaging of POPC and hybrid-vesicles

The vitrified POPC and POPC: polymer vesicles were used for cryo-transmission electron microscopy (Cryo-TEM). The plunging procedure was used to prepare the vesicles for cryo-TEM investigations. The temperature and humidity were maintained while plunging using an EM GP grid plunger (Leica, Wetzlar, Germany). The thin film of samples was prepared by dispersing the samples on EM-grids coated with a holey carbon film (C-flat, Protochips Inc., Raleigh, NC) and the excess solvents were removed. The verification of materials, below 108 K, was performed by rapidly plunging the grids into liquid ethane and the temperature was maintained just above the freezing point. The specimens were examined using a Libra 120 transmission electron microscope (Carl Zeiss Microscopy GmbH, Oberkochen, Germany), operating at 120 kV. The microscope was equipped with a Gatan 626 cryotransfer (Gatan, Las Poitas, USA) system and a BM-2 k-120 dual-speed on-axis SSCCD camera (TRS, Moorenweis, Germany).

The size distribution profile of cyro-TEM images presented in the **Figure S19** and compared with the size determined *via* DLS. The size profile was calculated using Image SP Viewer software.

Visualization of hybrid giant unilamellar vesicles (GUVs)

Hybrid giant unilamellar vesicles (GUVs) were prepared according to the established protocol for hybrid-GUVs.[4] In summary, a mixture of POPC and the polymer $(EO)_2P_{48}A$_**Co**_5% at a molar ratio of 95:5, along with 0.1 mol% of the fluorescent lipid analog Rh-DPPE (Lissamine rhodamine B-1,2-dipalmitoyl-sn-glycero-3-phosphoethanolamine), was dissolved in a chloroform solution (2:1, v/v) at a total lipid concentration of 10 mg/ml. This solution was

used for GUV preparation. Imaging of GUVs was performed using confocal fluorescence microscopy on an inverted LSM980 microscope (Carl Zeiss, Jena, Germany) at room temperature with a 40× 1.2 NA C-Apochromat water immersion objective. Excitation was achieved with a 561 nm diode laser, and fluorescence emission was detected using the AiryScan detector.

A$\beta_{1\text{-}40}$ fibrillation kinetic assays the presence of POPC and POPC: polymer vesicles via ThT

To investigate the influence of hybrid-vesicles on A$\beta_{1\text{-}40}$ aggregation the freshly prepared monomeric A$\beta_{1\text{-}40}$ peptide was incubated with the hybrids. All vesicles were freshly prepared and later on, used for A$\beta_{1\text{-}40}$ fibrillation assays within 10 hr. ThT dye ThT (λ_{ex} = 450 nm and λ_{em} = 480 nm) was used to track the aggregation progress while the interaction of the peptide with the vesicles. The peptide monomers were prepared by dissolving the peptide in 150 mM NaCl supplemented 50 mM phosphate buffer (pH 7.4) then the peptide solution sonicated for two minutes and subsequently centrifuged for two hrs at 10000 rpm. The supernatant layer of the solution was collected which contain A$\beta_{1\text{-}40}$ monomers. The UV-Vis spectroscopy was used to confirm the concentration of freshly prepared monomeric peptide using an absorption extinction co-efficient (ε) of 1490 M^{-1} cm^{-1} at 280 nm. A 200 μL solution of POPC or hybrid-vesicles was mixed physically with 10 μM A$\beta_{1\text{-}40}$ monomers and 10 μM ThT. 150 μL of the mixture was placed in 3 individual wells of a 96-well plate to investigate the influence of each hybrid-vesicles on A$\beta_{1\text{-}40}$ aggregation. The established agitation protocol was used to shake the mixture until the fibrillation completion.[4] A FLUOstar Omega plate reader (from BMG Labtech) was employed to track the change of ThT fluorescence from the bottom upon interactions of hybrid-vesicles with the A$\beta_{1\text{-}40}$ peptide while aggregation progression. Each normalized (0 to 1) individual fibrillation kinetics was fitted using the previously reported fitting function to determine lag time, t_{lag} (initial aggregation phase duration) and half time $t_{1/2}$ (when 50% monomeric peptide converted to fibrils).[5]

Mean of three normalized kinetics for both POPC and hybrid-vesicles was imported in the open-access online platform Amylofit.[6] The Amylofit platform possessed several mathematical models to either extract the mechanism of A$\beta_{1\text{-}40}$ aggregation or determine the microscopic steps of A$\beta_{1\text{-}40}$ aggregation altered by the interactions between the hybrids and peptide. The models correlate the interactions quantitatively using rate laws like

k_n, k_+ and k_2 for the rate constants of primary nucleation, elongation and secondary nucleation. Among the range of adopted models, only two models described the interactions of vesicles with the $A\beta_{1-40}$ quite nicely. One was unseeded secondary nucleation dominated and another one was unseeded fragmentation and secondary nucleation dominated.

Secondary structure determination *via* circular dichroism (CD) spectroscopy

Secondary structures of the $A\beta_{1-40}$ aggregates, upon interactions between hybrid-vesicles and $A\beta_{1-40}$ peptide, were monitored using a CD spectrometer (Jasco Corp., J-1500). The aggregated samples were placed within the 1 mm quartz cuvette and the CD spectra of the samples after ThT assays were recorded from 260 to 200 nm. The secondary structures of the native $A\beta_{1-40}$ peptide aggregates and in the presence of POPC vesicles devoid of polymers were also recorded for comparison. All spectra were the accumulation of ten (10) scans and recorded at 25 °C. The spectra were smoothened via integrated smoothening function of the spectrometer. The individual spectra were further imported in the BeStSel (Beta Structure Selection) algorithm and the secondary structures were estimated (see **Table S3**).[7]

Transmission electron microscopy (TEM) of $A\beta_{1-40}$ aggregates

The morphology of the aggregated $A\beta_{1-40}$ peptide was explored using TEM imaging. A very diluted 5 μL aggregated sample after kinetic assays were placed on 200 mesh size copper (Cu) grids coated with Formvar/Carbon film. After three minutes of incubation, the excess liquid was bloted and the grids were washed three times with 30 μL water each time. Then the grids were stained with 8 μL uranyl acetate solution and the excess uranyl acetate was bloted completely after one minute incubation. The grids were dried overnight before imaging. A Zeiss EM 900 transmission (Carl Zeiss Microscopy GmbH, Jena, Germany) electron microscope operating at 80 kV was used for imaging. The images were further analyzed using ImageSP Viewer software.

Liquid-State Nuclear Magnetic Resonance (NMR) Spectroscopy of POPC and hybrid-vesicles

The NMR spectra were recorded using Varian Gemini 200 (400 MHz) or Varian Unity Inova 500 (500 MHz) NMR spectrometer from Agilent Technologies Germany GmbH & Co. KG, Waldbronn, Germany. The ^{1}H-NMR spectra of polymers as well as ^{1}H- and ^{31}P-NMR spectra of hybrid-vesicles were measured using the same instrument in deuterated chloroform ($CDCl_3$). The spectra were analysed via MestReNova software (version 11.0.4-18998).

Electrospray Ionization Time-of-Flight Mass Spectroscopy (ESI-TOF MS)

ESI-TOF MS measurements were conducted using A Fucos Micro TOF from Bruker Daltonics. 1-2 mg of sample was dissolved in a mixture of HPLC grade THF and methanol (100: 1) at a concentration of 1 mg mL^{-1} with sodium iodide (NaI) salt. A 180 μLh^{-1} flow rate was maintained while injecting the sample and a 4.5 kV acceleration voltage was applied to capture the spectra in the positive mode. The Data Analysis 4.2 software from Bruker Daltonics was used to interpret the data.

Matrix-Assisted Laser Desorption Ionization Time-of-Flight Mass Spectroscopy (MALDI-TOF MS) of polymers

A Bruker Autoflex III Smart beam MALDI-TOF MS spectrometer was used to analyze the polymers. The device has a λ = 337 nm nitrogen laser and can operate in both linear and reflection modes. The reflection mode was suitable to analyse the polymers. Trans-2-[3-(4-tert-butylphenyl)-2-methyl-2 propenylidene] malononitrile (DCTB) was the matrix to prepare the samples for the measurement. The solutions of matrix, polymers and sodium trifluoroacetate (NaTFA) were prepared at a concentration of 20 mg mL-1 in THF separately. A ratio of 100: 10: 1 was used to prepare the mixture of polymer, matrix and salt. The mixture was used for the MALDI-TOF MS measurements. The Data Analysis 4.2 software from Bruker Daltonics was employed to interpret the MALDI-TOF MS measurements.

Matrix-Assisted Laser Desorption Ionization Time-of-Flight Mass Spectroscopy (MALDI-TOF MS) of Hybrid-vesicles

The hybrid-vesicles were dried and MALDI-TOF MS of hybrid-vesicles (HVs) were performed following the similar protocol used for the polymers.

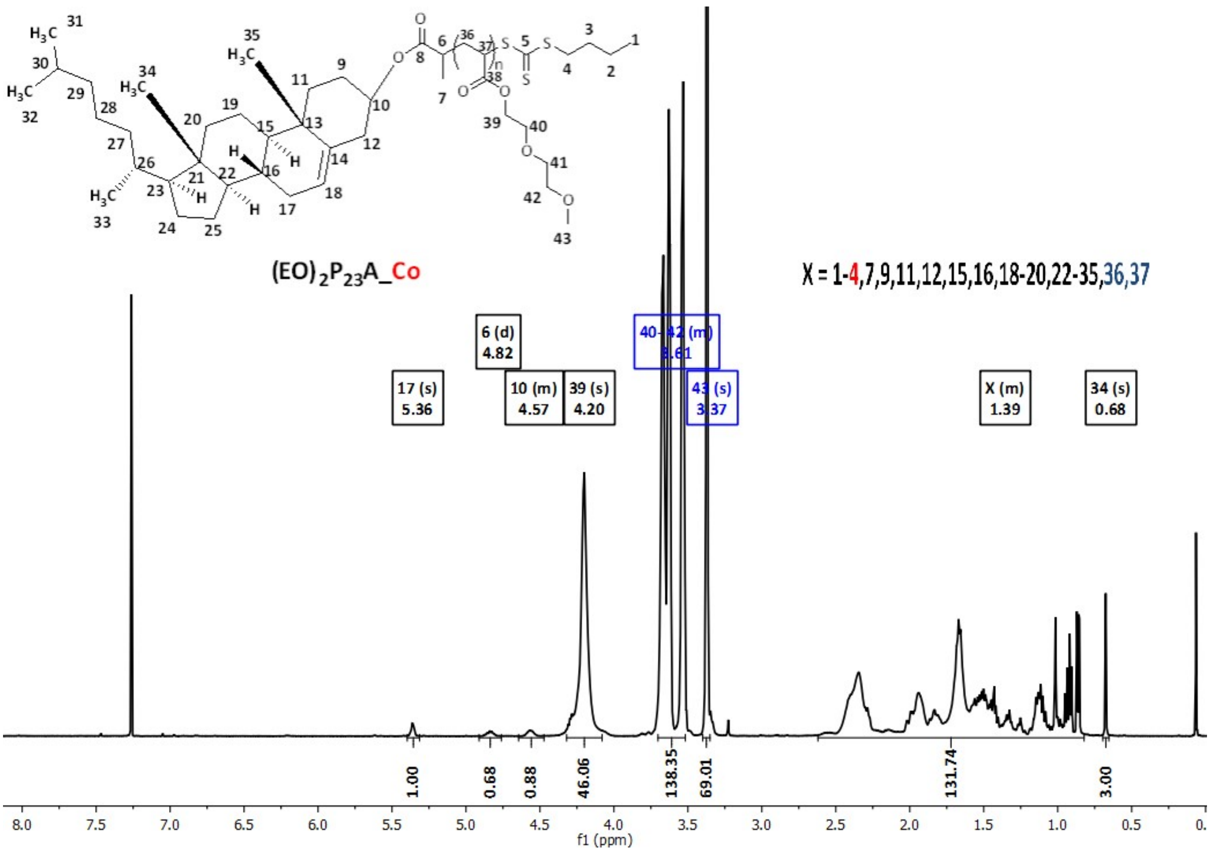

Figure S1: ^{1}H-NMR spectrum of $(EO)_2P_{23}A$_**Co** in deuterated chloroform ($CDCl_3$) at 500 MHz.[1]

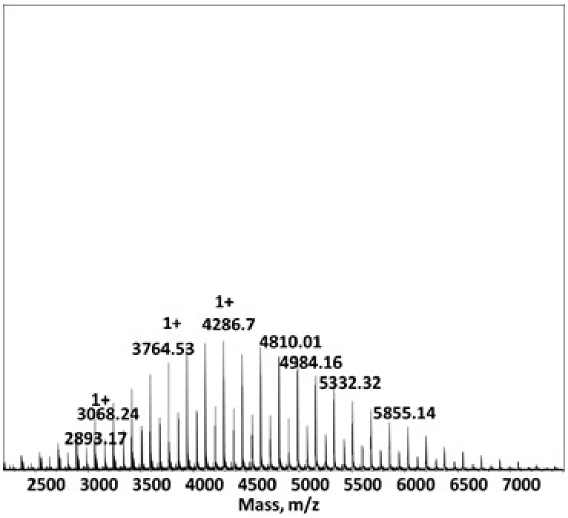

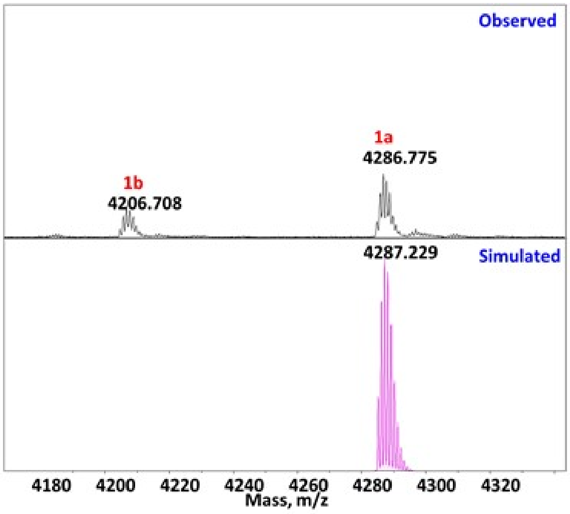

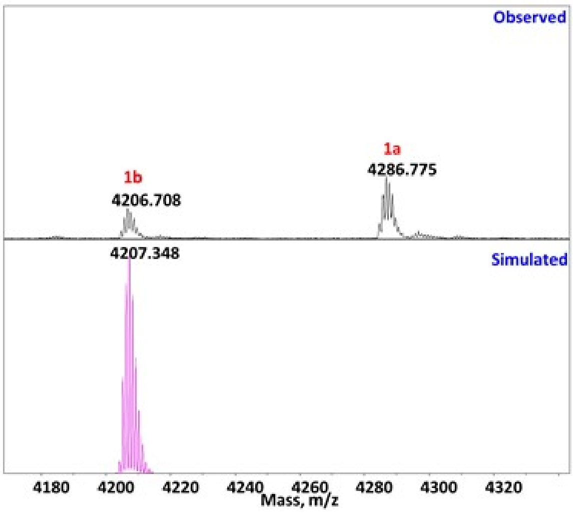

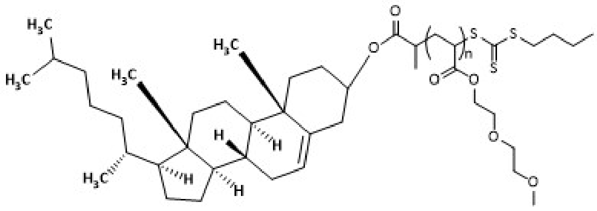

$(EO)_2P_{23}A\_Co$

$[M] = C_{30}H_{49}O_2(C_8H_{14}O_4)_nC_5H_9S_3$

Calculated mass for n = 23

1a = $[M+Na]^+$ = 4287.2

1b = $[M-C_4H_9+Na]^+$ = 4207.3

Figure S2: MALDI-TOF MS spectrum of $(EO)_2P_{23}A$_**Co**. Full spectrum and expansion of the molecular ion isotopic peaks with observed (upper) and simulated (lower) signals with assignments[1]

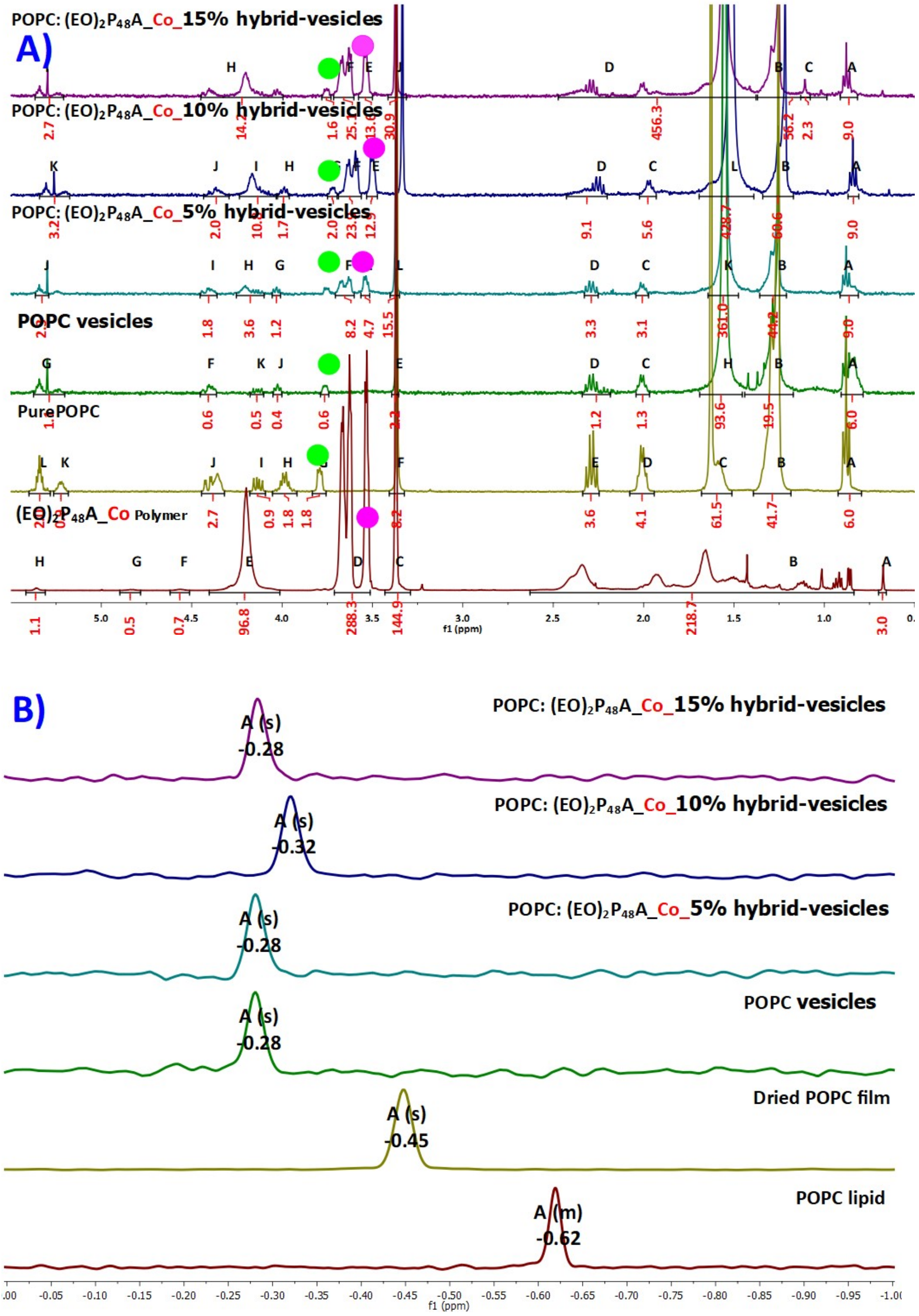

C) Sample Name	POPC position (ppm)	$(EO)_2P_{23}A$_Co position (ppm)	Added polymer molar percentage	^{1}H-NMR determined molar percentage
POPC: $(EO)_2P_{23}A$_Co_5%	4.00	3.54	5 %	7.6 %
POPC: $(EO)_2P_{23}A$_Co_10%	3.76	3.54	10 %	11.8 %
POPC: $(EO)_2P_{23}A$_Co_15%	3.76	3.54	15%	15.1 %

Figure S3: (**A**) ^{1}H-and (**B**) ^{31}P-NMR of the hybrid-vesicles in deuterated chloroform ($CDCl_3$) and the calculated polymer composition from ^{1}H-NMR in hybrid-vesicle (**C**).

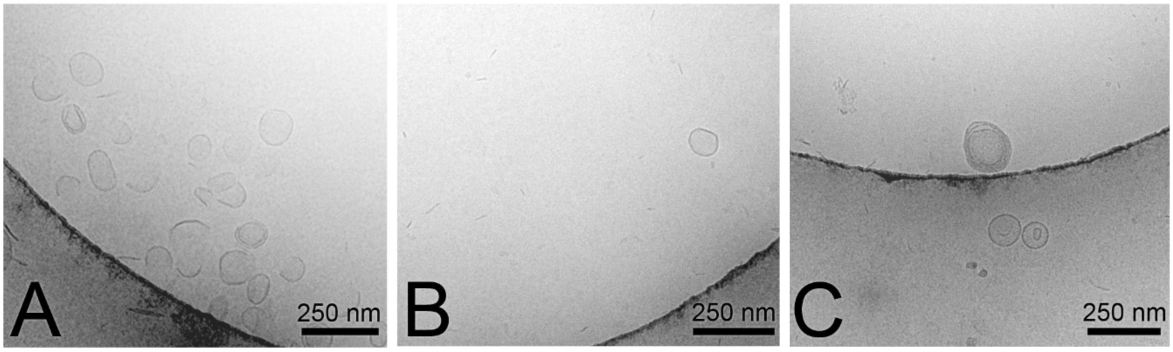

Figure S4: Cryo-TEM images of hybrid-vesicles containing **A**) $(EO)_2P_{19}A$_**Hy**_15%, **B**) $(EO)_2P_{23}A$_**Co**_10% and **C**) $(EO)_3P_{10}A$_**Co**_10% polymers embedded in POPC lipid. The scale bar of the images is 250 nm.

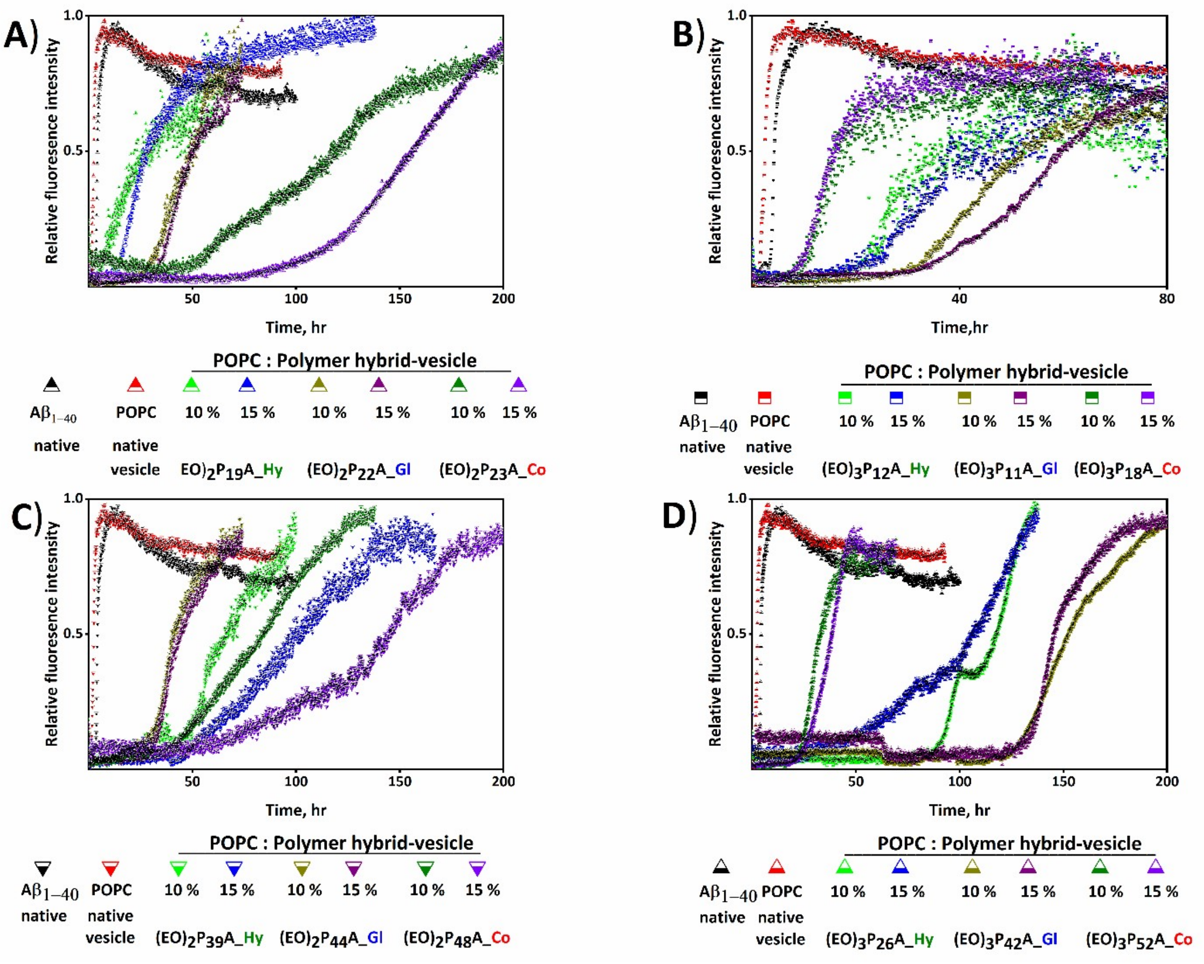

Figure S5: Influence of hybrid-vesicles on $A\beta_{1-40}$ aggregation. Panel **A** and **B** represent the influence of comparable low molecular weight polymers bearing hybrid-vesicles, whereas panels **C** and **D** are for the high molecular weight polymers bearing hybrid-vesicles. The fibrillation kinetics monitored using ThT fluorescence intensity in a 50 mM Na_2HPO_4 buffer supplemented with 150 mM NaCl at pH 7.4. The black error bars of all normalized kinetics indicate the standard deviation among the three independent measurements. The **Hy**, **Gl** and **Co** represent the hexadecyl, glyceryl and cholesteryl anchor groups.

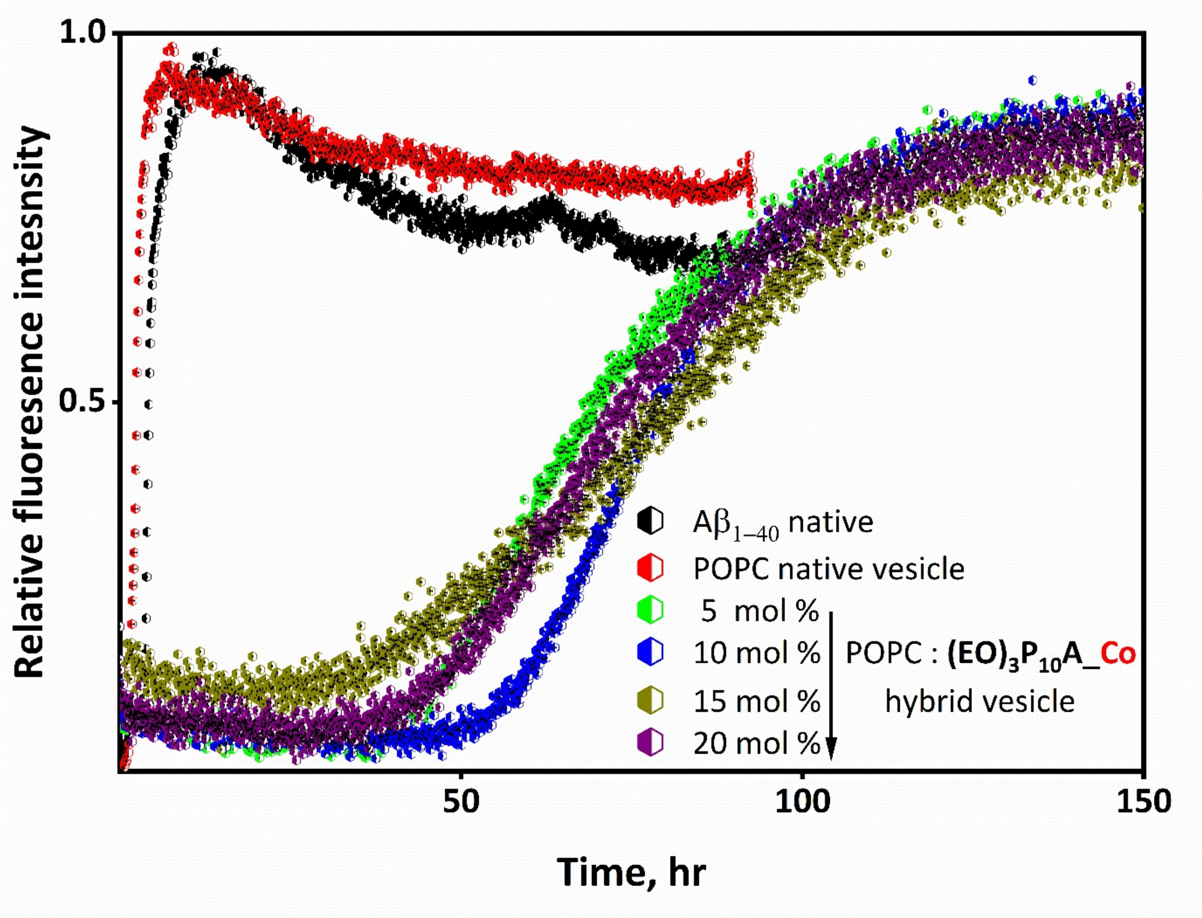

Figure S6: Influence of hybrid-vesicles bearing 5-20 mol% of $(EO)_3P_{10}A$_Co polymers on $A\beta_{1\text{-}40}$ aggregation. The normalized ThT fluorescence with errors is presented and compared with the influence of $A\beta_{1\text{-}40}$ aggregation in the presence of native POOC vesicles.

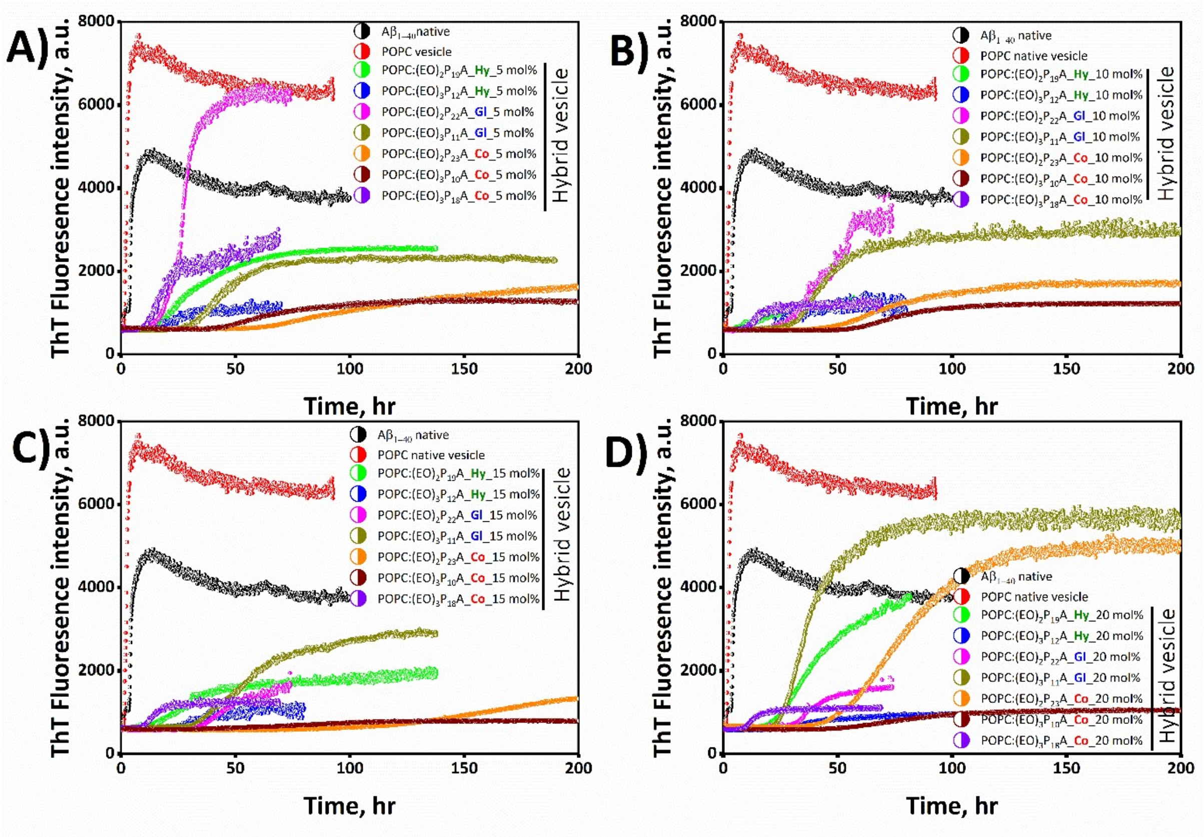

Figure S7: Non-normalized aggregation ThT monitored aggregation kinetics in the presence of hybrid-vesicles. Comparable low molecular weight polymers in variable molar percentages up to 20 mol% incorporated in the hybrids. Mean of three individual kinetics marked with respective colors for each hybrid-vesicles. The errors of three individual kinetics are marked with black bars. The $A\beta_{1\text{-}40}$ fibrillation kinetics of $A\beta_{1\text{-}40}$ alone and in the presence of POPC presented in each panel to compare the influence of hybrid-vesicles.

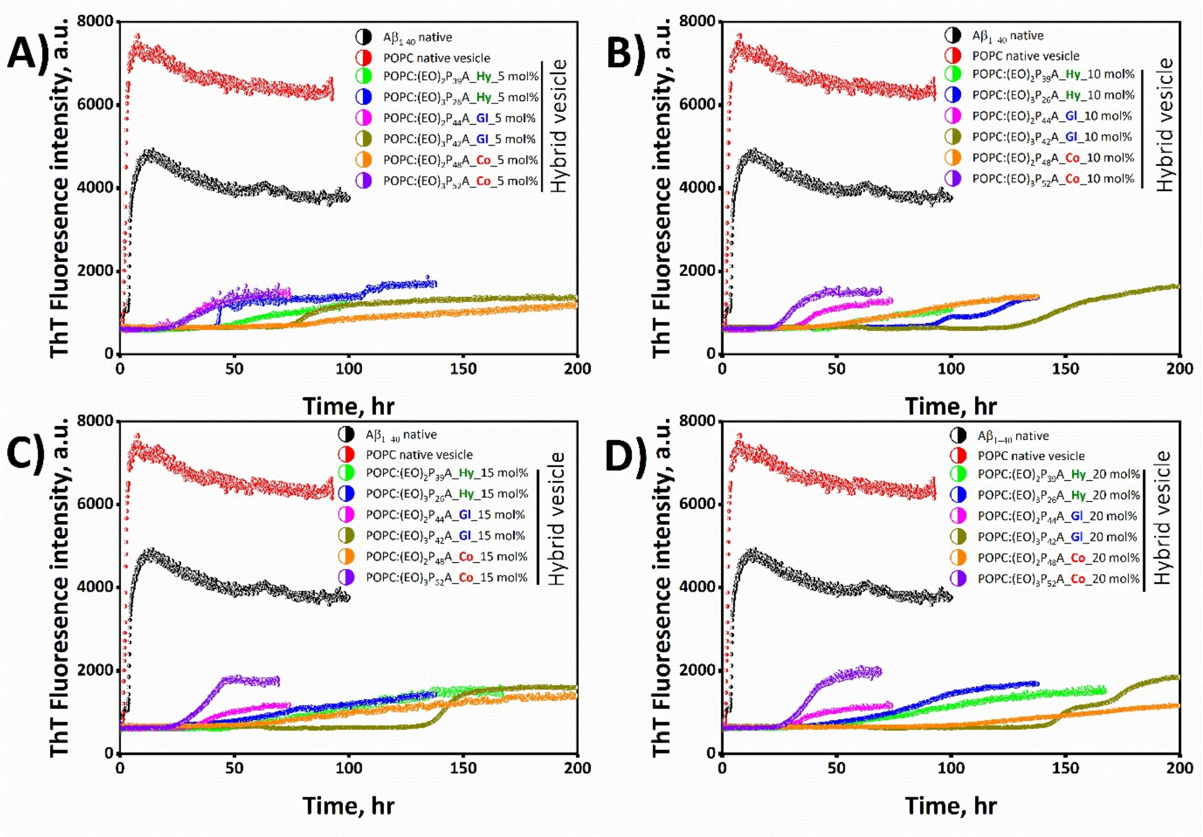

Figure S8: Aggregation kinetics of the A$\beta_{1\text{-}40}$ peptide in the presence hybrid-vesicles bearing comparable high molecular weight polymers. A variable molar percentage of up to 20 mol% incorporated in the hybrids. Three individual kinetics are averaged (presented with respective colors) for each hybrid-vesicles including the A$\beta_{1\text{-}40}$ peptide alone and POPC vesicles without any polymers incorporation. All kinetic assays were conducted in 50 mM Na_2HPO_4 buffer solution supplemented with 150 mM NaCl at pH 7.4 and 37 °C.

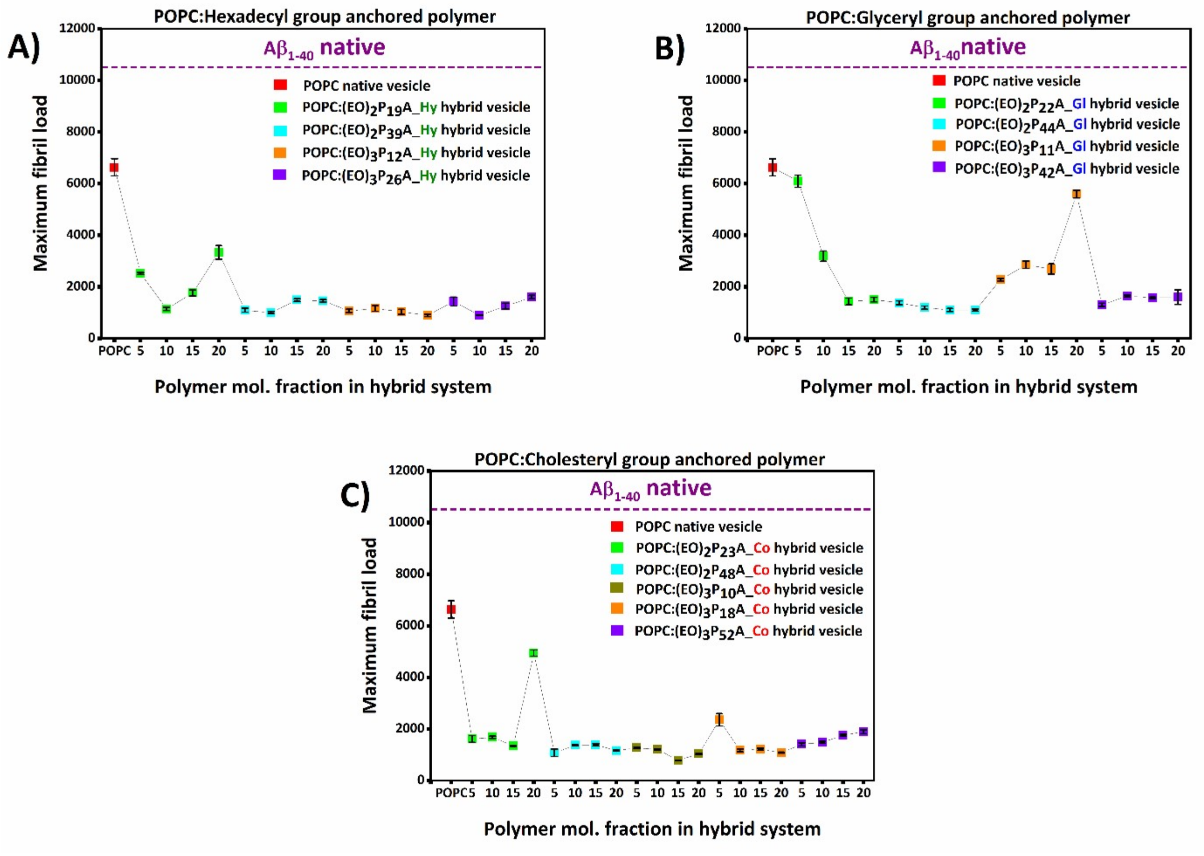

Figure S9: Fibril load in the aggregates estimated from the maximum of non-normalized ThT fluorescence in the presence of POPC and hybrid-vesicles. A$\beta_{1\text{-}40}$ peptide fibril load in the absence of vesicles presented to compare hybrid-vesicles influence on the amount of fibril formation. The standard deviation of three individual kinetics indicated by black error bars. Fibril load of hexadecyl (**Hy**), glyceryl (**Gl**) and cholesteryl (**Co**) group anchored polymers bearing hybrid-vesicles are presented in **A**, **B** and **C** respectively.

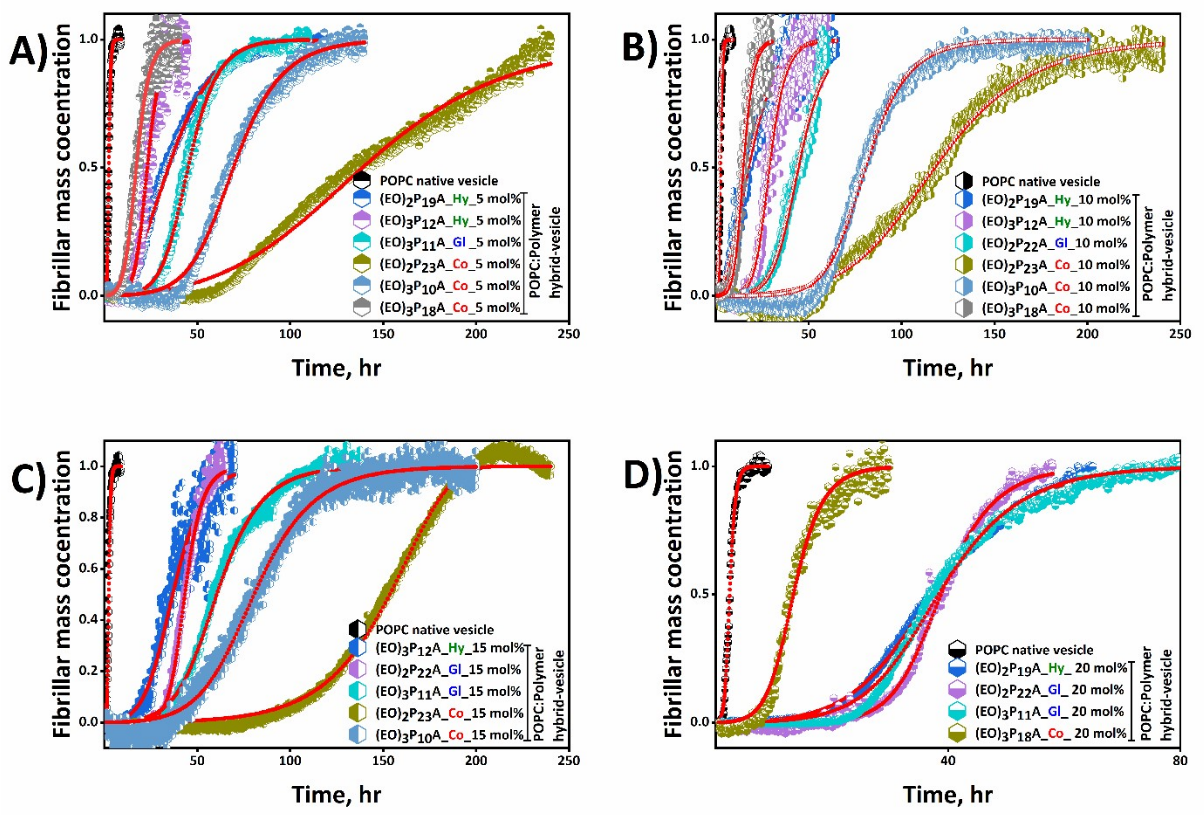

Figure S10: Time-resolved ThT fluorescence kinetics fitted in Amylofit.[6] The kinetic profiles revealed the influence of POPC and hybrid-vesicles on $A\beta_{1-40}$ fibrillation. Mean of three normalized individual kinetics for each measurement imported in the online platform of Amylofit. The secondary nucleation dominated unseeded model unveiled the changes of fibrillation in the presence of POPC and most hybrid-vesicles presented in the figure, except the hybrids bearing $(EO)_3P_{10}A$_**Co**_15 mol% polymer (panel **C**), which was fitted with the fragmentation and secondary nucleation dominated unseeded model. The rate constants, associated with the particular microscopic steps of $A\beta_{1-40}$ fibrillation, obtained from the fitting are presented in detail in **Table S2**.

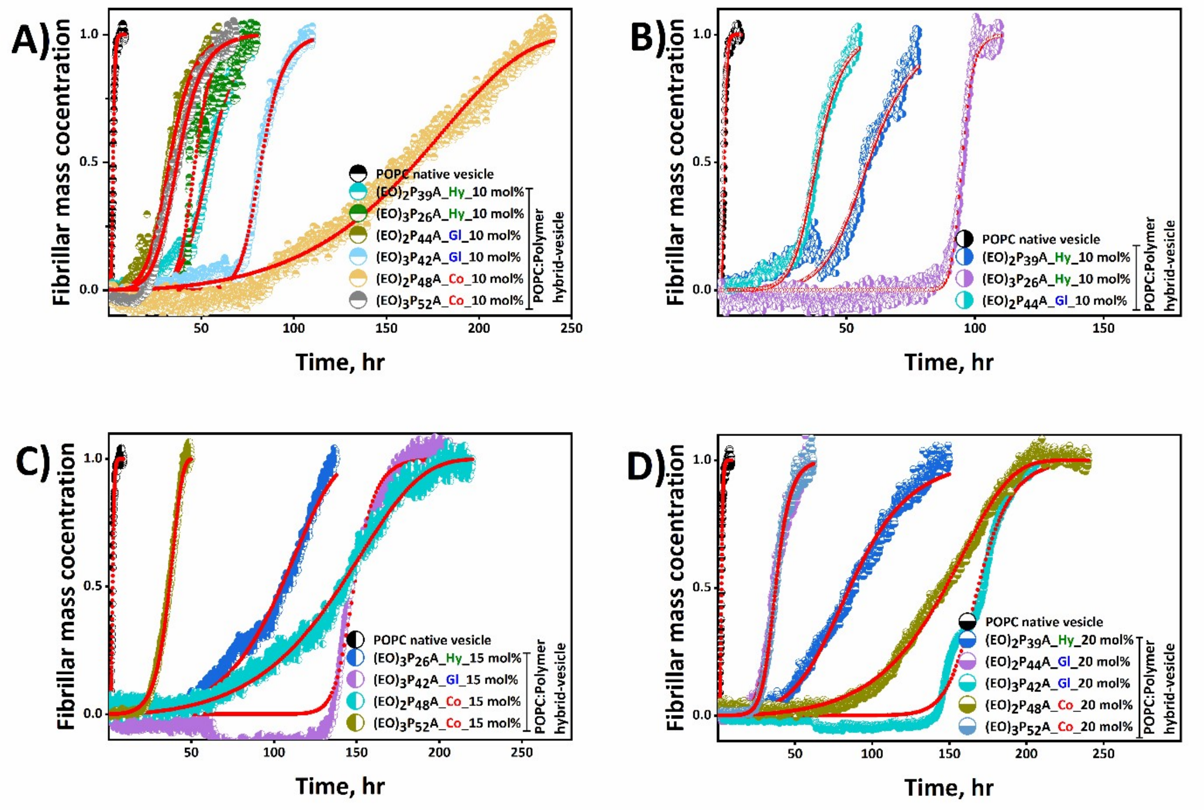

Figure S11: Normalized time-evolved ThT fluorescence kinetics fitted in Amylofit to reveal the impact of hybrid-vesicles on Aβ_{1-40} fibrillation and the influence of hybrids compared with the POPC vesicles. The unseeded secondary nucleation dominated model fitted the majority of fibrillation kinetics in the presence of hybrid-vesicles presented in the Figure. Another unseeded fragmentation and secondary nucleation dominated model suitably fitted the fibrillation kinetics in the presence of $(EO)_3P_{26}A$_**Hy**_15 mol%, $(EO)_2P_{48}A$_**Co**_10 mol%, $(EO)_2P_{48}A$_**Co**_15 mol%, $(EO)_2P_{48}A$_**Co**_20 mol%, $(EO)_3P_{52}A$_**Co**_15 mol% polymer embedded hybrid-vesicles. The rate constants of specific microscopic steps, involved in Aβ_{1-40} fibrillation, determined from the fitting using models presented in **Table S2**.

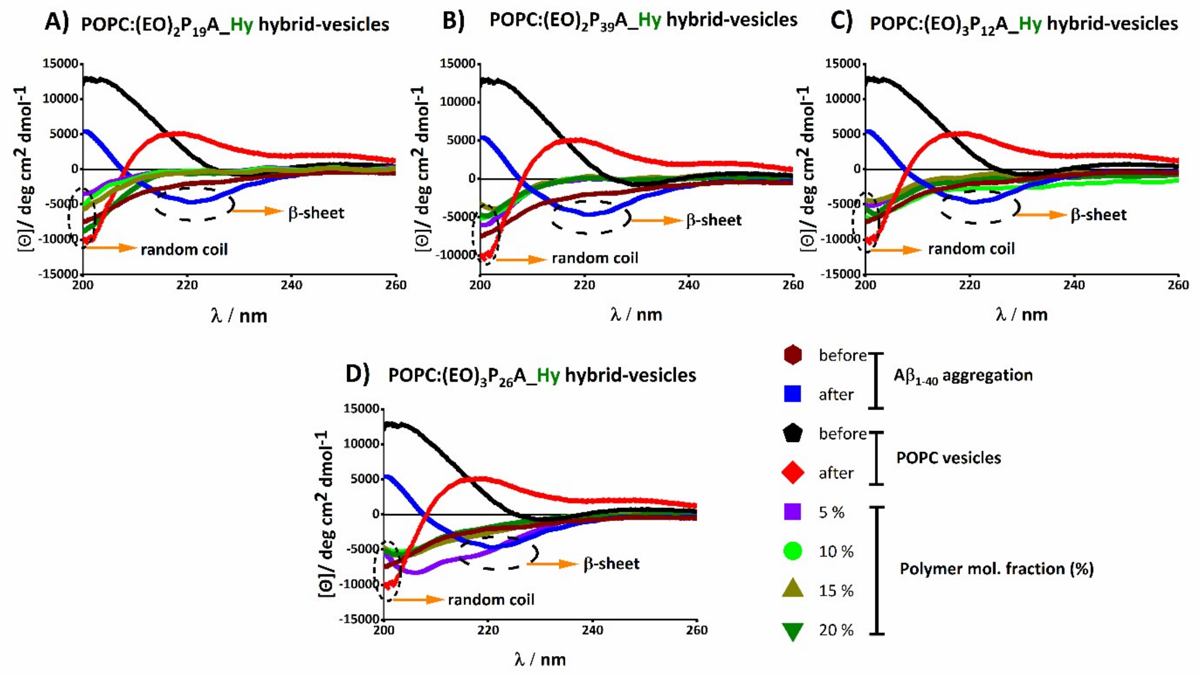

Figure S12: CD spectra of $A\beta_{1\text{-}40}$ either in the presence of pure POPC and hybrid-vesicles, compared to the non-aggregated $A\beta_{1\text{-}40}$ peptide after ThT dependent fibrillation kinetic assay. The CD-spectra represent $A\beta_{1\text{-}40}$ peptide fibrillation in the presence of **A**) $(EO)_2P_{19}A$_Hy, **B**) $(EO)_2P_{39}A$_Hy, **C**) $(EO)_3P_{12}A$_Hy and **D**) $(EO)_3P_{26}A$_Hy polymers bearing POPC: polymer vesicles. Inset of the plot presents the amount (up to 20 mol%) of embedded polymers in hybrid-vesicles.

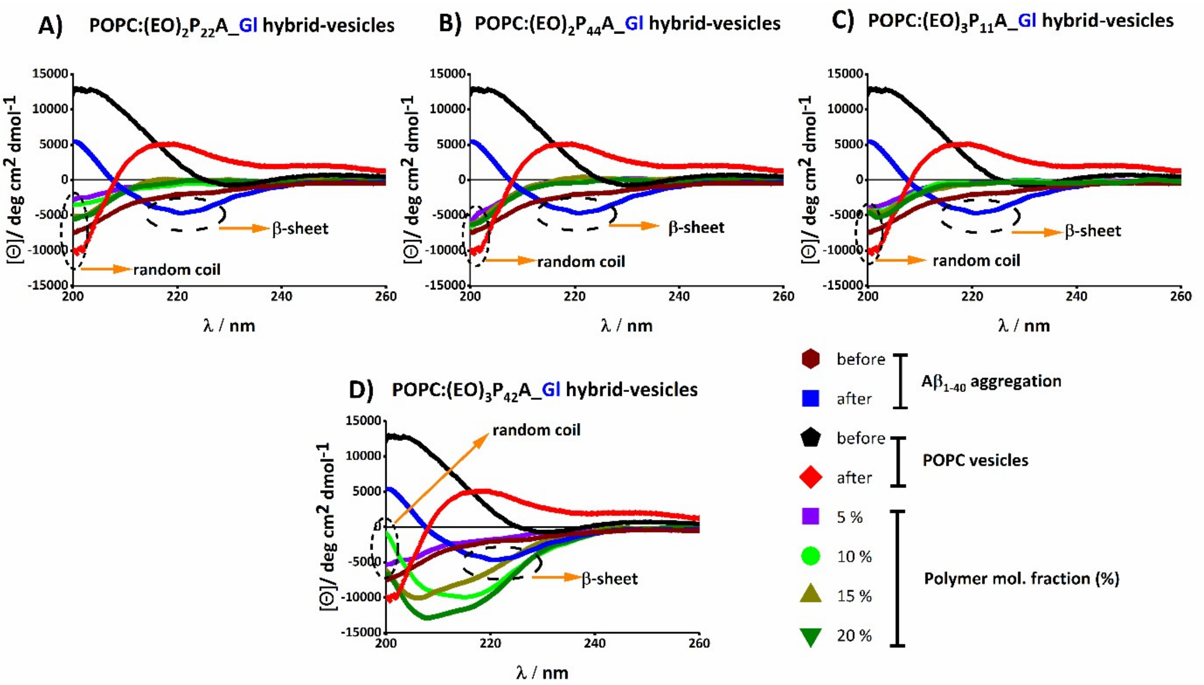

Figure S13: CD spectra of A$\beta_{1\text{-}40}$ peptide in the presence of **A**) $(EO)_2P_{22}A$_**Gl**, **B**) $(EO)_2P_{44}A$_**Gl**, **C**) $(EO)_3P_{11}A$_**Gl** and **D**) $(EO)_3P_{42}A$_**Gl** polymers (up to 20 mol%) bearing hybrid-vesicles. the CD-spectra of native A$\beta_{1\text{-}40}$ peptide and POPC devoid of polymers presented to compare. Polymer mole fractions in hybrids are present in the plot inset.

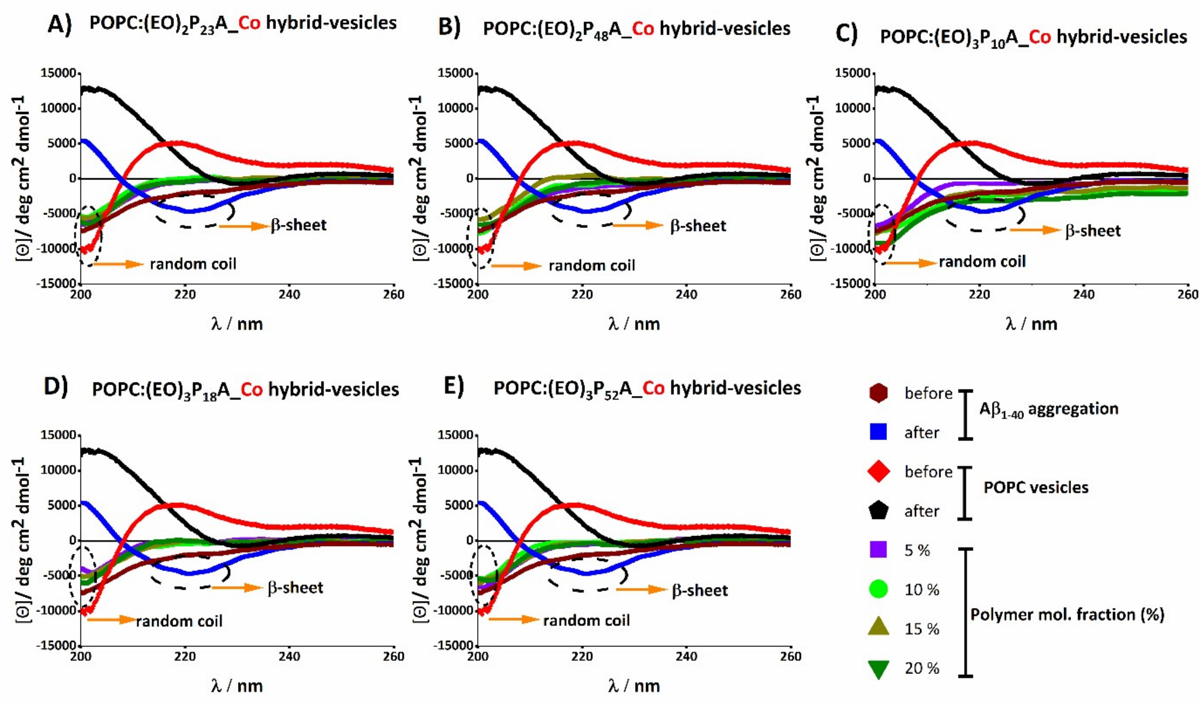

Figure S14: The A$\beta_{1\text{-}40}$ peptide aggregated in the presence of **A**) $(EO)_2P_{23}A$_**Co**, **B**) $(EO)_2P_{48}A$_**Co**, **C**) $(EO)_3P_{10}A$_**Co**, **D**) $(EO)_3P_{18}A$_**Co** and **E**) $(EO)_3P_{52}A$_**Co** polymers incorporated hybrid-vesicles. CD spectra of A$\beta_{1\text{-}40}$ peptide represent the respective polymers bearing hybrids. The amount of polymers in the hybrids is shown in the inset of the plot.

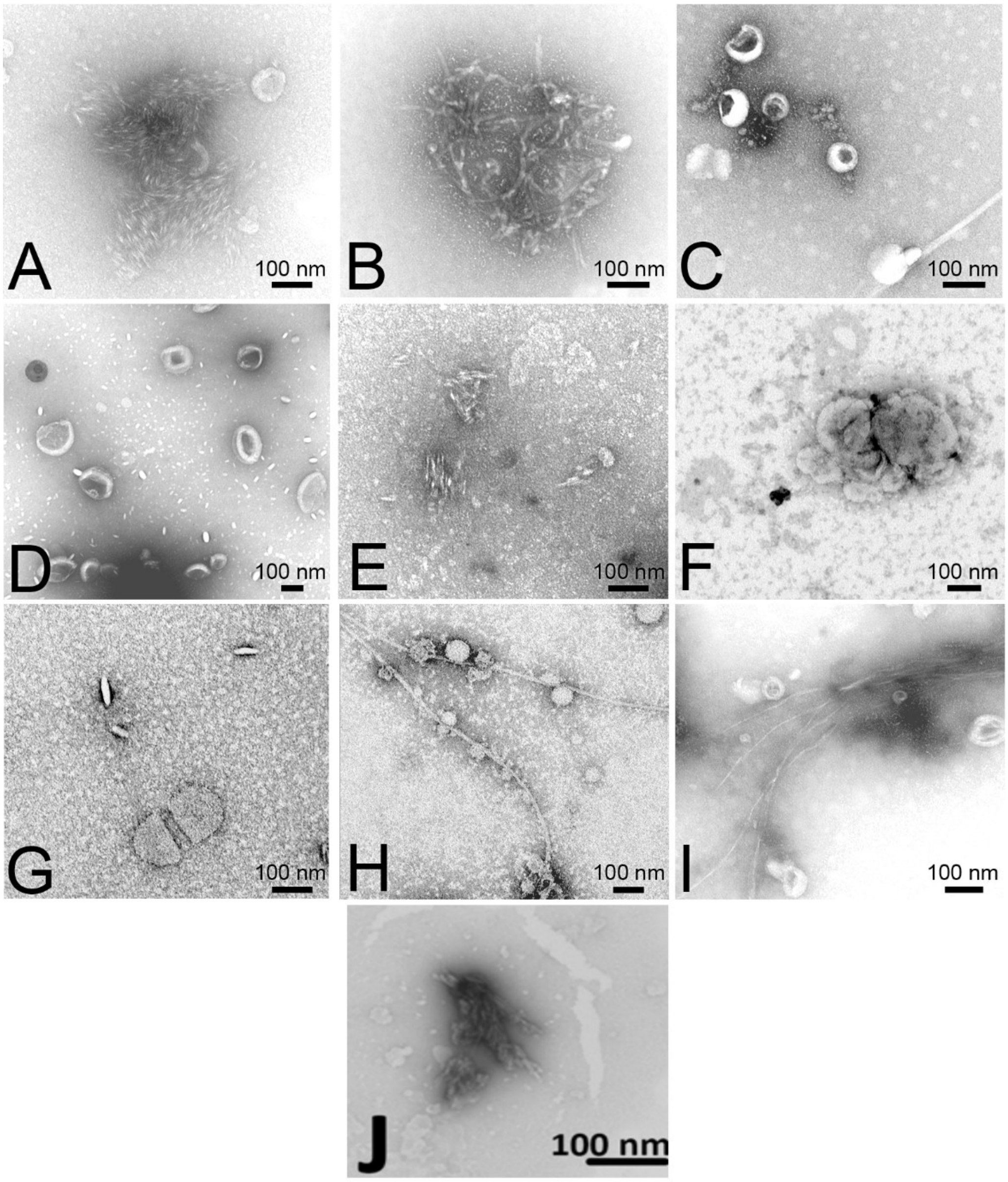

Figure S15: TEM images of A$\beta_{1\text{-}40}$ fibrils in the presence of **A**) POPC vesicles and hybrid-vesicles bearing **B**) $(EO)_2P_{19}A$_**Hy**_5%, **C**) $(EO)_2P_{19}A$_**Hy**_15%, **D**) $(EO)_3P_{26}A$_**Hy**_15%, **E**) $(EO)_2P_{22}A$_**Gl**_20%, **F**) $(EO)_3P_{11}A$_**Gl**_15%, **G**) $(EO)_3P_{11}A$_**Gl**_20%, H) $(EO)_2P_{23}A$_**Co**_10%, **I**) $(EO)_2P_{48}A$_**Co**_20% and **J**) $(EO)_2P_{48}A$_**Co**_20% polymers in POPC lipid. The scale bar of the images is 250 nm.

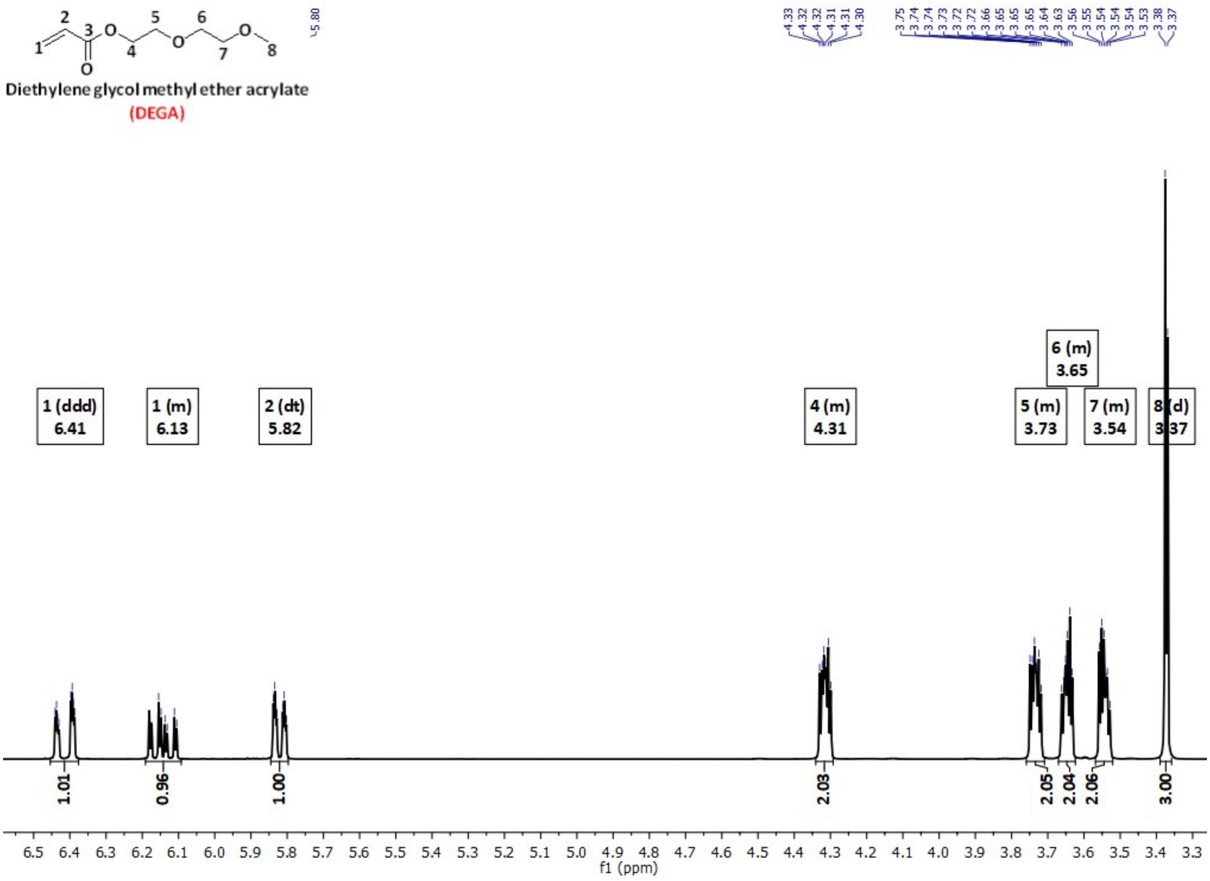

Figure S16: ^{1}H-NMR spectrum of diethylene glycol methyl ether acrylate (DEGA) in deuterated chloroform ($CDCl_3$) at 500 MHz.[1]

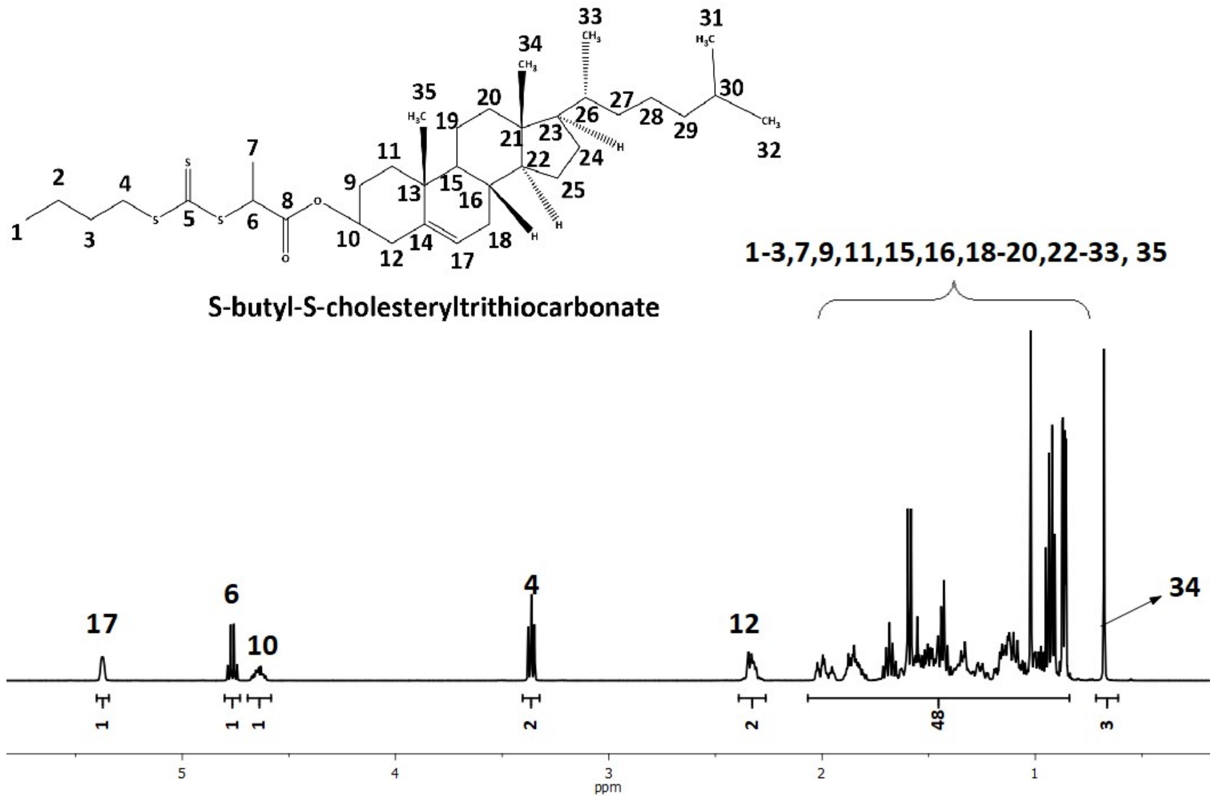

Figure S17: ^{1}H-NMR spectrum of S-butyl-S-cholesteryltrithiocarbonate in deuterated chloroform ($CDCl_3$) at 500 MHz.[1]

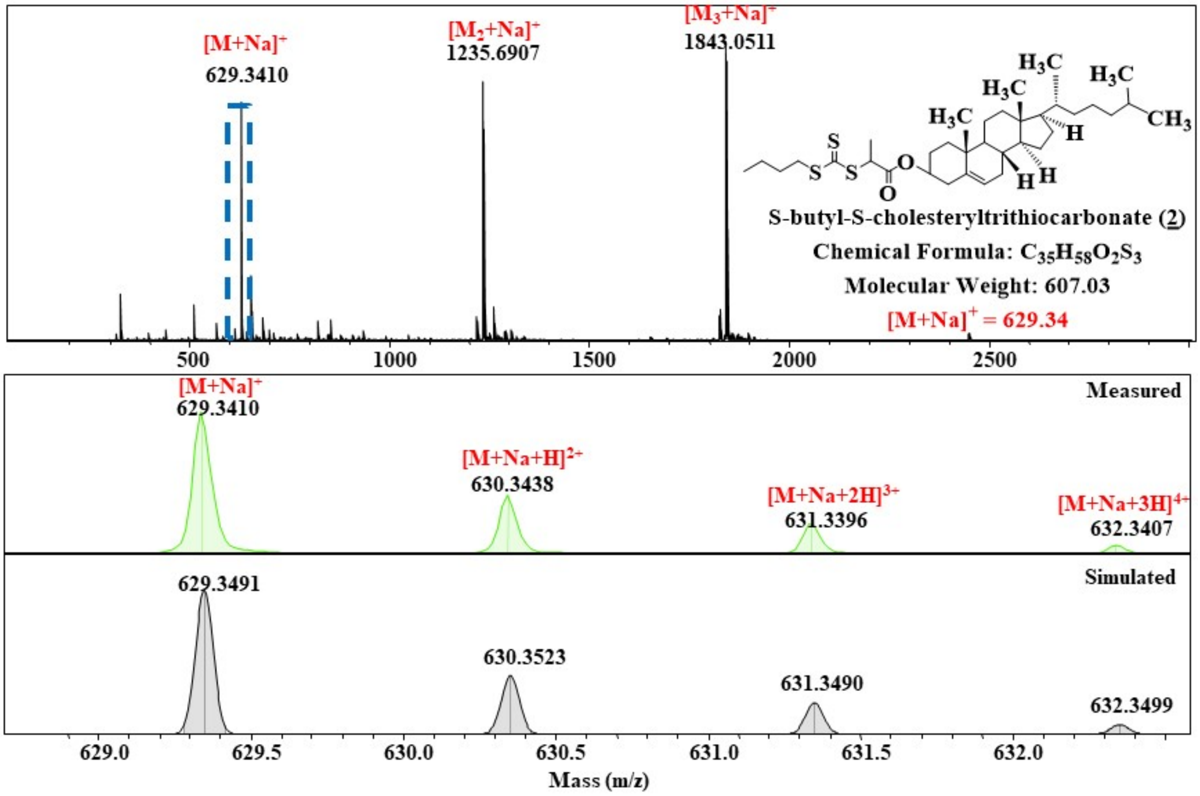

Figure S18: ESI-TOF MS spectra of S-butyl-S-cholesteryltrithiocarbonate. (A) Full spectrum, (B) measured and simulated isotopic signals of the marked section.[1]

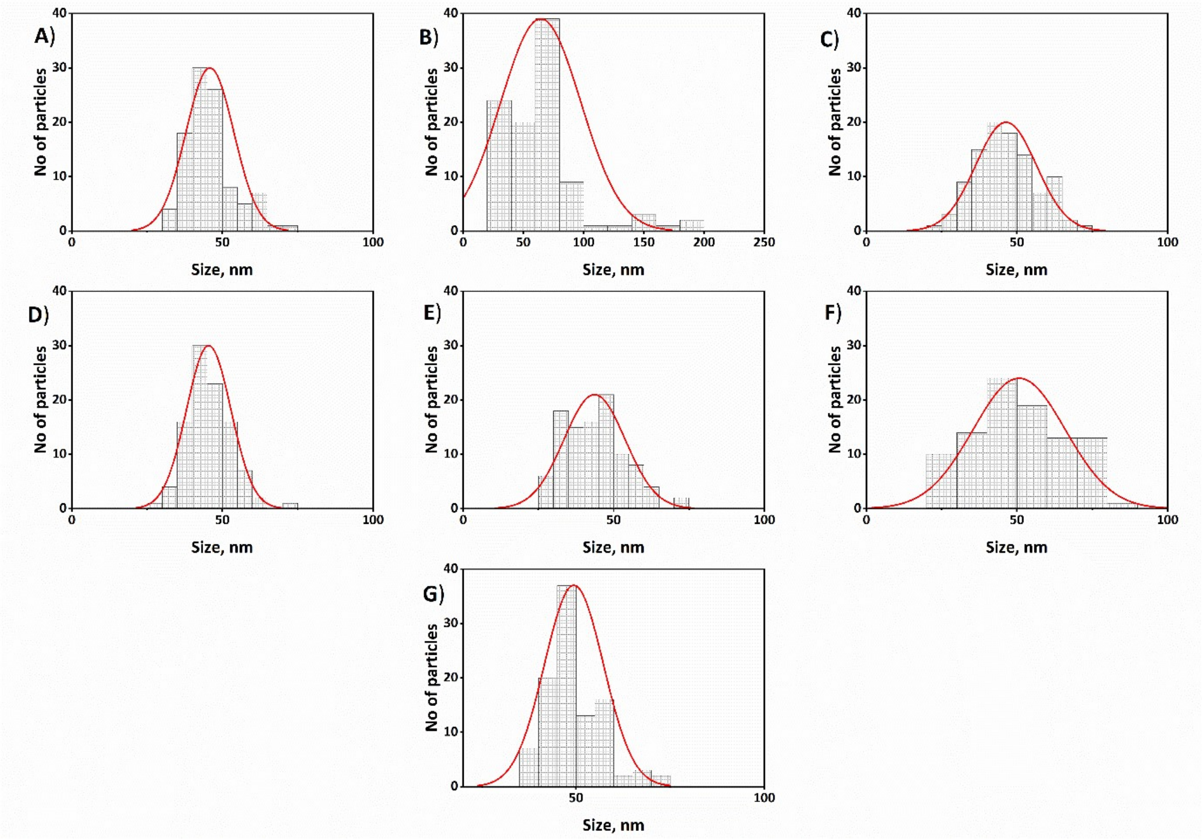

Hybrid vesicles	D_h (nm)	Size EM (Avg. 100 particles) nm		
		Mean (nm)	Min. (nm)	Max. (nm)
POPC:$(EO)_3P_{26}A$_**Hy**_20%	58.0	45.9	33	72.4
POPC:$(EO)_3P_{11}A$_**Gl**_5%	103.4	64.5	20.9	198.7
POPC:$(EO)_3P_{42}A$_**Gl**_5%	103.3	46.4	24.7	70.6
POPC:$(EO)_3P_{42}A$_**Gl**_20%	86.5	45.5	26.8	72.8
POPC:$(EO)_2P_{48}A$_**Co**_10%	78.7	43.7	25.1	72.8
POPC:$(EO)_3P_{18}A$_**Co**_10%	81.3	50.7	20.5	80.8
POPC:$(EO)_3P_{52}A$_**Co**_10%	80.1	49.4	35	73.1

Figure S19: Size distribution profile of cyro-TEM images hybrid vesicles embedded with (A) **$(EO)_3P_{26}A$_Hy_20%**, (B) $(EO)_3P_{11}A$_**Gl**_5%, (C) $(EO)_3P_{42}A$_**Gl**_5%, (D) $(EO)_3P_{42}A$_**Gl**_20%, (E) $(EO)_2P_{48}A$_**Co**_10%, (F) $(EO)_3P_{18}A$_**Co**_10% and (G) $(EO)_3P_{52}A$_**Co**_10% polymers in POPC. 100 hybrid vesicles size were calculated from cyro-TEM images and tabulated. Hybrid-vesicles were prepared in same buffer used in ThT assays. Hybrid vesicles size compared to hydrodynamic diameter (D_h).

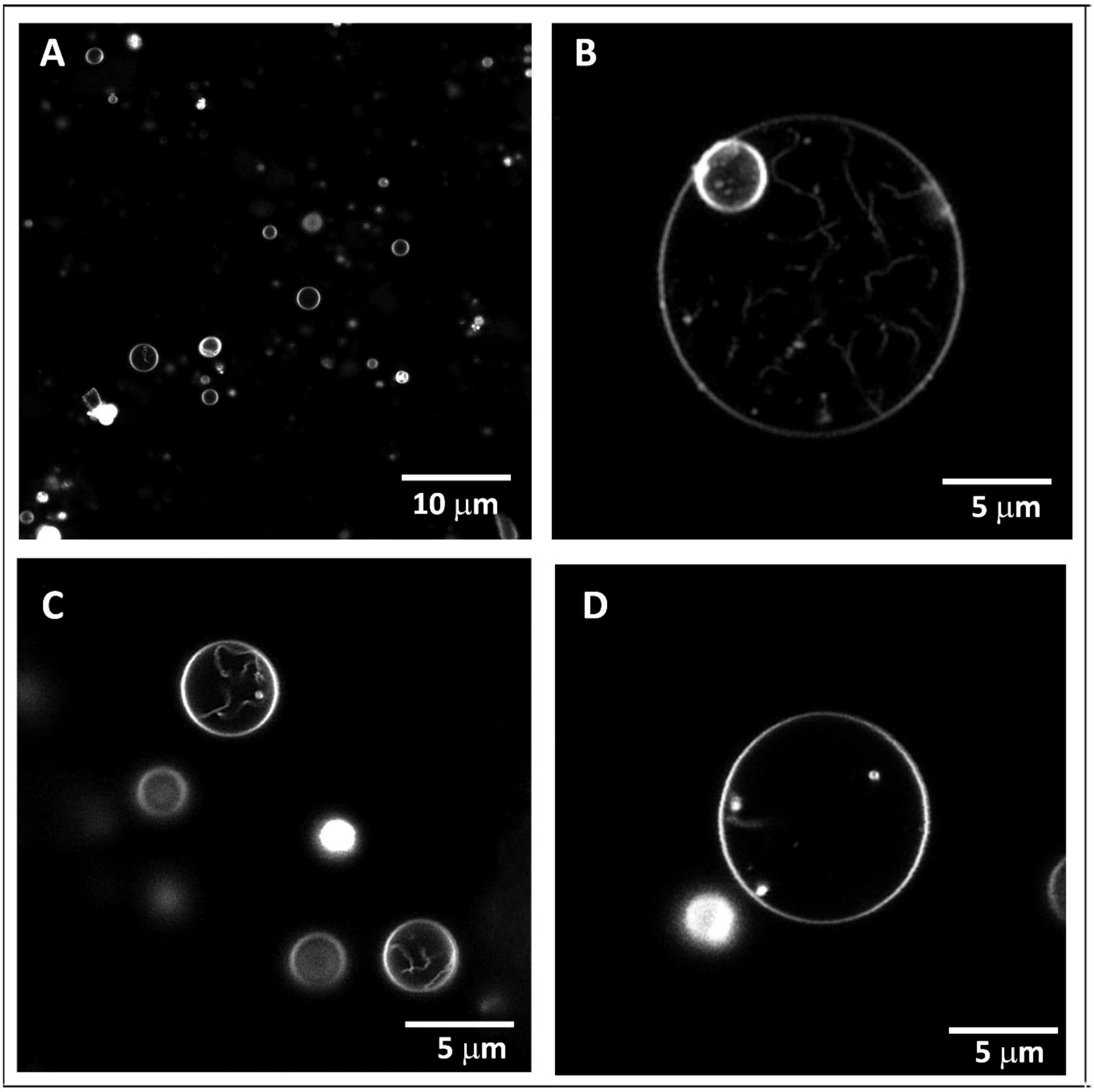

Figure S20: Lipophilic fluorescent tracer Rh-DPPE was used to visualize the membrane heterogeneities of pure POPC GUVs and hybrid-GUVs at room temperature using Confocal microscopy. Panel (**A** and **B**) represent pure POPC GUVs and a single POPC GUV. Panel (**C** and **D**) depict the hybrid-GUVs composed of POPC and 5 mol% $(EO)_2P_{48}A$_**Co** polymer. The images display a uniform distribution of the fluorescent dye Rh-DPPE, in pure DOPC and hybrid-GUVs, confirming the homogeneous nature of the lipid bilayer in both pure POPC GUVs and hybrid-GUVs. Scale bars: (**A**) 10 µm, (**B**) 5 µm, (**C**) 5 µm, (**D**) 5 µm.

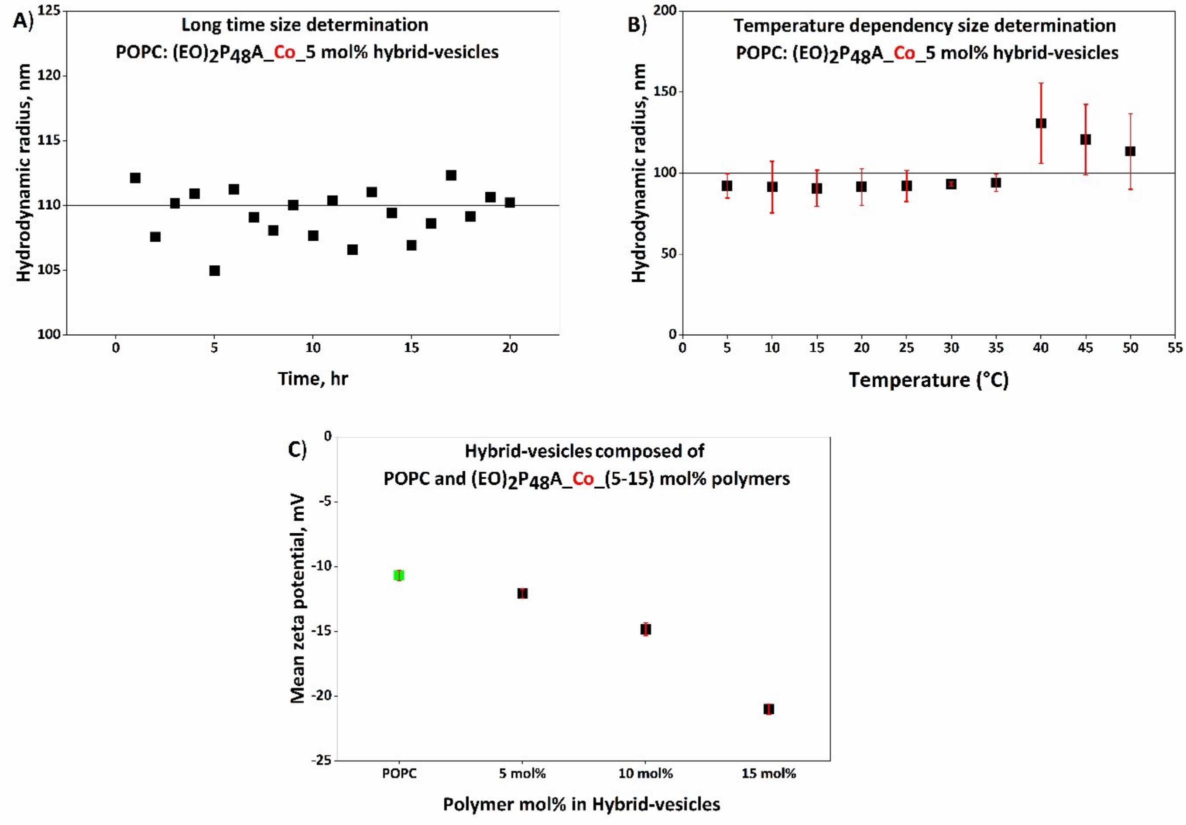

Figure S21: The size of the hybrid-vesicles, composed of POPC and 5 mol% $(EO)_2P_{48}A$_**Co** polymer, was monitored over an extended period (A) and across a range of temperatures (B). Zeta potential (ζ) of hybrid-vesicles bearing 5 mol% to 15 mol% $(EO)_2P_{48}A$_**Co** polymers measured at room temperature. Three repetitive measurements for each hybrid-vesicles averaged and presented with standard deviation (±).

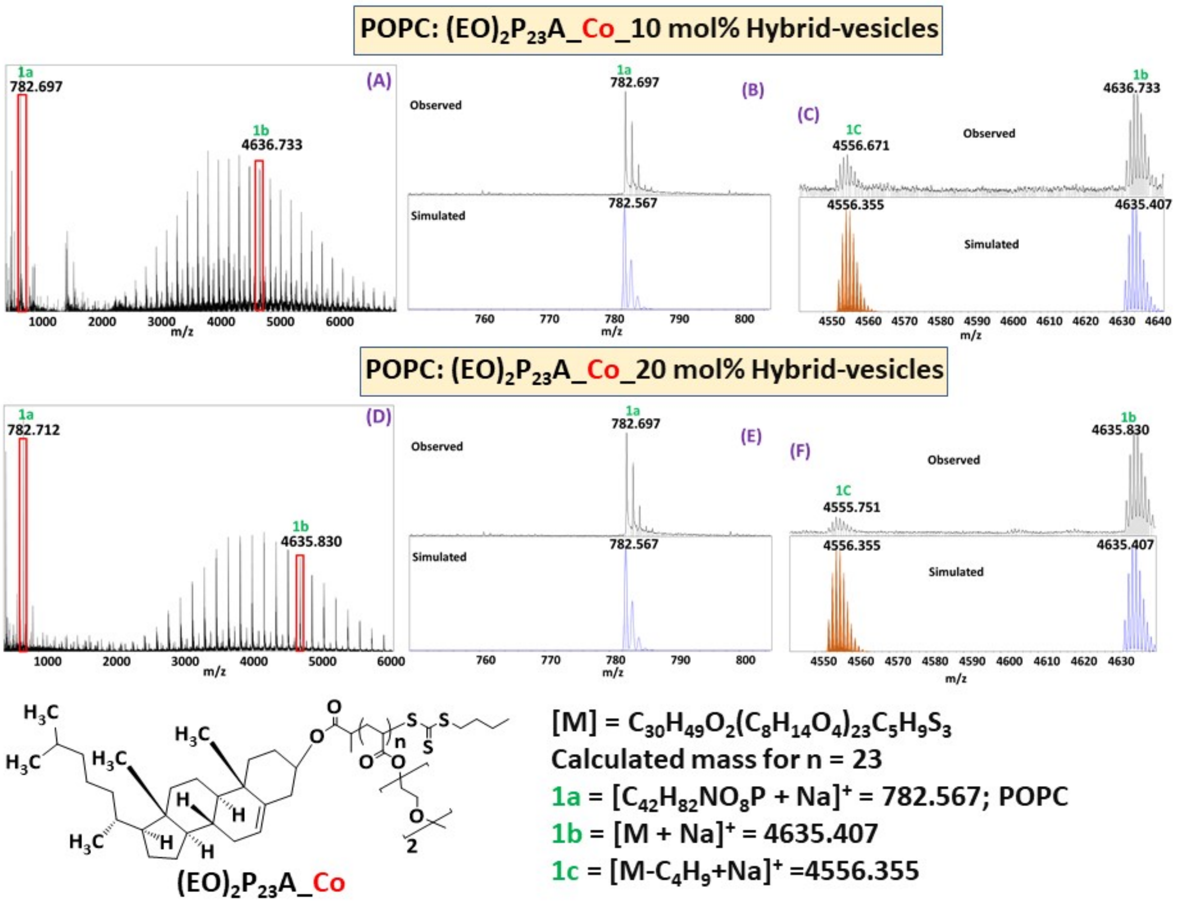

Figure S22: MALDI-TOF MS spectrum of hybrid-vesicles (HVs) comprised of POPC and $(EO)_2P_{23}A$_Co_10 mol% (upper panel) and $(EO)_2P_{23}A$_Co_20 mol% (lower panel).

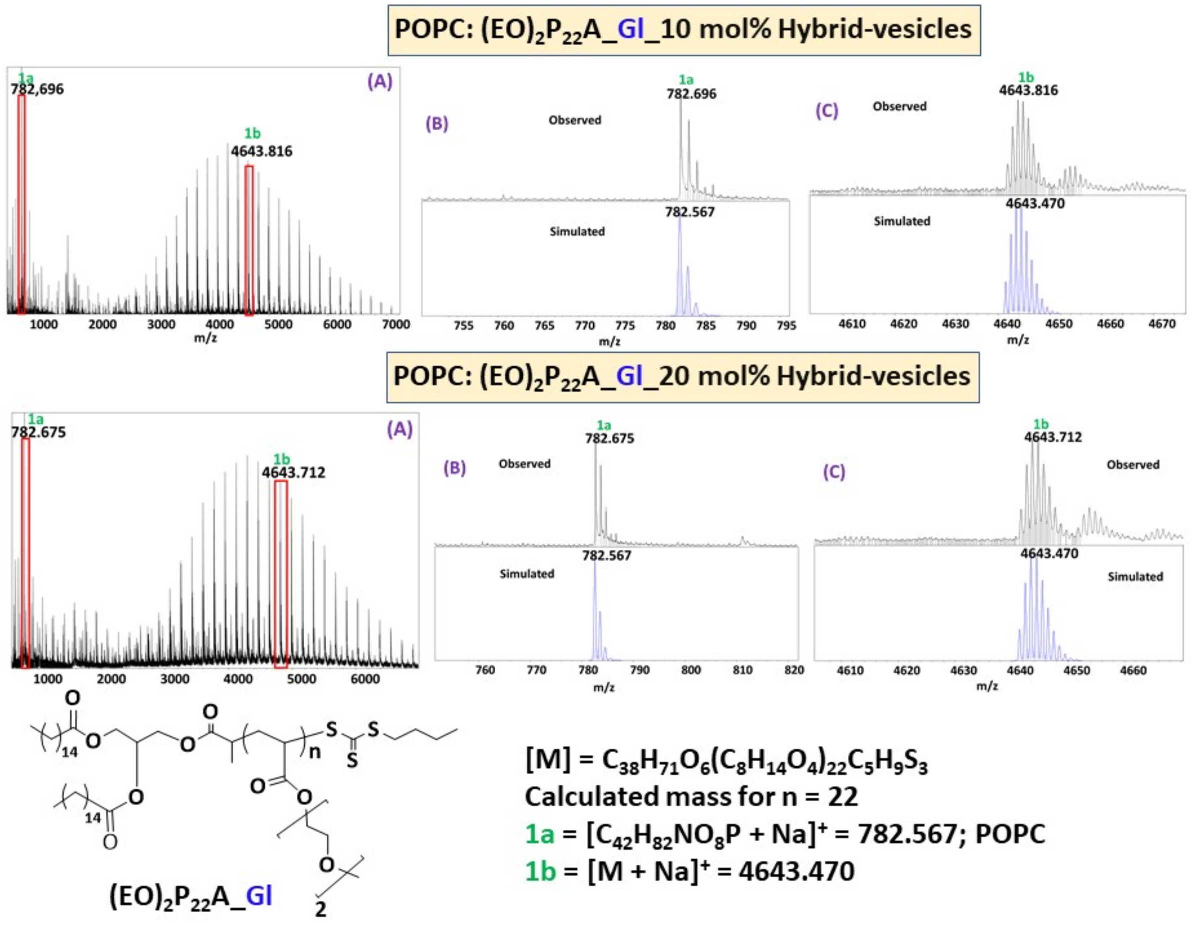

Figure S23: MALDI-TOF MS spectrum of hybrid-vesicles (HVs) comprised of POPC and $(EO)_2P_{22}A$_Gl_10 mol% (upper panel) and $(EO)_2P_{22}A$_Gl_20 mol% (lower panel).

Table S1: Summary of POPC and hybrid-vesicles including size and composition of the vesicles and their influence on Aβ_{1-40} fibrillation. The lag (t_{lag}) and half time ($t_{1/2}$) of Aβ_{1-40} aggregation in the presence of POPC and hybrid-vesicles are obtained by fitting the ThT fluorescence kinetics using a piecewise fitting function. Mean t_{lag} and $t_{1/2}$ of three individual kinetics with corresponding standard deviation (±) are presented here. Vesicles preparation and fibrillation kinetics of Aβ_{1-40} was conducted in 50 mM Na_2HPO_4 pH 7.4 buffer supplemented with 150 mM NaCl. Kinetic investigations were performed at 37 °C.

Entry	Peptide	Core lipid and conc. of lipid (mM)	Embedded Polymer	Anchored group	Polymer incorporation in mol%	Size[a] (nm)	PDI[a]	Half time ($t_{1/2}$), hr	Lag time (t_{lag}), hr
**1**	Aβ_{1-40}							4.3 ± 0.2	3.7 ± 0.02
**2**	Aβ_{1-40}	POPC (1.5 mM)						2.3 ± 0.5	1.9 ± 0.01
**3**	Aβ_{1-40}				5	83 4	0.10	33.9 ± 2.7	14.8 ± 0.1
**4**			$(EO)_2P_{19}A$_Hy		10	94.5	0.15	25.9 ± 3.3	6.6 ± 0.4
**5**					15	103.0	0.19	27.7 ± 0.0	13.9 ± 0.1
**6**					20	94.1	0.17	39.6 ± 1.5	22.2 ± 0.1
**7**					5	76.5	0.17	68.6 ± 1.3	42.9 ± 0.2
**8**			$(EO)_2P_{39}A$_Hy		10	78.1	0.18	71.1 ± 4.2	47.2 ± 0.3
**9**					15	83.9	0.18	105.3 ± 5.3	48.6 ± 0.4
**10**					20	93.1	0.19	97 ± 8.9	47.3 ± 0.4
**11**				Hexadecyl	5	192.3	0.17	34.2 ± 1.7	14.2 ± 0.5
**12**			$(EO)_3P_{12}A$_Hy	(Hy)	10	190.4	0.19	38.7 ± 5.1	19.9 ± 0.4
**13**					15	144.8	0.20	37.3 ± 5.2	24.6 ± 0.3
**14**					20	196.5	0.10	44.9 ± 7.5	26.9 ± 0.2
**15**					5	62.6	0.10	72.5 ± 33.2	64.8 ± 0.1
**16**			$(EO)_3P_{26}A$_Hy		10	71.6	0.20	112.8 ± 15.8	104.0 ± 0.1
**17**					15	60.6	0.20	98.9 ± 27.5	80.5 ± 0.2
**18**					20	58.0	017	86.8 ± 15.6	57.1 ± 0.2
**19**					5	121.3	0.10	27.7 ± 0.5	17.5 ± 0.1
**20**			$(EO)_2P_{22}A$_Gl		10	153.5	0.18	49.01± 4.2	27.6 ± 0.1
**21**					15	160.5	0.16	48.2 ± 8.6	35.7 ± 0.1
**22**					20	158.1	0.16	40.7 ± 1.7	30.8 ± 0.1
**23**					5	75.2	0.17	34.6 ± 1.7	25.7 ± 0.2
**24**			$(EO)_2P_{44}A$_Gl		10	76.0	0.23	41.3 ± 2.3	32.4 ± 0.1
**25**					15	73.9	0.23	44.7 ± 1.7	31.7 ± 0.1
**26**					20	73.2	0.26	39.4 ± 3.8	29.5 ± 0.1
**27**		POPC (1.5 mM)		Glyceryl	5	103.4	0.21	44.6 ± 4.4	30.8 ± 0.1
**28**			$(EO)_3P_{11}A$_Gl	(Gl)	10	131.4	0.20	54.5 ± 7.0	33.1 ± 0.2
**29**					15	155.4	0.13	60.1 ± 12.1	42.0 ± 0.1
**30**					20	189.2	0.23	38.3 ± 3.5	25.8 ± 0.1
**31**					5	103.3	0.25	95.1 ± 15.2	75.9 ± 0.2
**32**			$(EO)_3P_{42}A$_Gl		10	81.0	0.21	151.3 ± 4.8	128.5 ± 0.1
**33**					15	85.4	0.10	150.7 ± 12.1	139.6 ± 0.1
**34**					20	86.5	0.20	167 ± 14	154.8 ± 0.1
**35**					5	82.4	0.10	143.2 ± 45.7	67.3 ± 0.3
**36**			$(EO)_2P_{23}A$_Co		10	87.3	0.10	113.2 ± 36.7	80.1 ± 0.3
**37**					15	102.0	0.15	160.2 ± 17.1	98.5 ± 0.3
**38**					20	93.5	0.11	97.7 ± 34.2	60.6 ± 0.1
**39**					5	79.2	0.23	152.3 ± 49.3	111.3 ± 0.3
**40**			$(EO)_2P_{48}A$_Co		10	78.7	0.13	84.4 ± 5.6	42.4 ± 0.3
**41**					15	62.1	0.22	136.4 ± 34.8	102.9 ± 0.2
**42**					20	54.7	0.17	148.8 ± 21.2	104.3 ± 0.3
**43**					5	153.2	0.10	69.3 ± 7.8	44.5 ± 0.2
**44**			$(EO)_3P_{10}A$_Co		10	85.4	0.17	78.4 ± 6.8	57.3 ± 0.1
**45**				Cholesteryl	15	176.4	0.15	76.7 ± 24.4	54.6 ± 0.3
**46**				(Co)	20	117.5	0.20	74.9 ± 10.2	45.9 ± 0.2
**47**					5	73.0	0.17	22.4 ± 2.0	9.2 ± 2.0
**48**			$(EO)_3P_{18}A$_Co		10	81.3	0.21	17.9 ± 0.8	9.2 ± 0.3
**49**					15	69.1	0.16	16.2 ± 2.2	9.4 ± 0.1
**50**					20	93.8	0.21	14.4 ± 0.9	8.9 ± 0.1
**51**					5	78.2	0.19	45.8 ± 8.4	24.1 ± 0.2
**52**			$(EO)_3P_{52}A$_Co		10	80.1	0.21	33.1 ± 1.3	22.9 ± 0.1
**53**					15	73.1	0.13	37.9 ± 3.7	26.13 ± 0.1
**54**					20	78.2	0.16	39.1 ± 1.7	27.5 ± 0.1

a) Size measured at 25 °C using DLS.

Table S2: Summary of rate constants estimated using mathematical models of Amylofit. Here, k_n, k_+ and k_2 are the primary nucleation, elongation and secondary nucleation rate constants for A$\beta_{1\text{-}40}$ aggregation. The combined rate constant k_+k_n for primary nucleation pathway, k_+k_2 for secondary nucleation pathway and k_-k_+ for fragmentation and elongation pathway. The M_o, n_c and n_2 represent the initial monomer concentration, primary and secondary nucleus size, respectively.

Entry	Lipid	Polymer name	Anchored Group	No of EO	Polymer incorporation in mol %	M_o [M]	k_+k_n [$M^{-2}hr^{-2}$]	n_c	k_+k_2 [$M^{-3}hr^{-2}$]	n_2	K_-k_+ [$M^{-1}hr^{-2}$]
1	POPC						1.7E10		3.42E18		
2					5		5.61E8		3.99E15		
3		$(EO)_2P_{19}A$_Hy			10		3.2E9		7.63E15		
4					15		8.57E8		5.7E15		
5					20		8.04E7		1.34E16		
6				2	5		4900000		1.35E16		
7		$(EO)_2P_{39}A$_Hy			10		7810000		9.66E15		
8					15		1.59E7		1.82E15		
9			Hexadecyl (Hy)		20		2.4E7		2.02E15		
10					5		4.8E7		6.22E16		
11		$(EO)_3P_{12}A$_Hy			10		2.73E7		4.25E16		
12					15		1.23E8		1.12E16		
13					20		4.74E7		1.52E16		
14				3	5		369000		3.57E16		
15		$(EO)_3P_{26}A$_Hy			10		7.7E-13		1.6E17		
16					15		1.66E7		8.35E14		0.0108
17					20		2.81E7		1,00E+15		0.00559
18					5		8360000		6.93E16		
19		$(EO)_2P_{22}A$_Gl			10		1.92E7		1.5E16		
20					15		824000		3.63E16		
21					20		2250000		3.82E16		
22				2	5		8.83E7		1.88E16		
23		$(EO)_2P_{44}A$_Gl			10	1E-6	5600000	2	3.16E16	2	
24					15		4340000		2.86E16		
25					20		1.82E7		2.44E16		
26			Glyceryl (Gl)		5		2.44E7		1.3E16		
27	POPC	$(EO)_3P_{11}A$_Gl			10		1.27E7		1.7E16		
28					15		2.56E7		5.53E15		
29					20		4.27E7		1.63E16		
30				3	5		1800		2.21E16		
31		$(EO)_3P_{42}A$_Gl			10		5600		4.31E15		
32					15		0.00776		1.98E16		
33					20		57.3		6.73E15		
34					5		1.93E7		4.57E14		
35		$(EO)_2P_{23}A$_Co			10		9960000		1.22E15		
36					15		2210000		8.29E14		0.0157
37					20		2.19E7		1.74E15		
38				2	5		8960000		2.47E14		0.0237
39		$(EO)_2P_{48}A$_Co			10		5.87E7		4.61E14		1.37E-4
40					15		1.21E7		3.62E14		0.00617
41			Cholesteryl (Co)		20		6740000		4.95E14		0.00138
42					5		1.48E7		4.72E15		
43		$(EO)_3P_{10}A$_Co			10		1940000		6.38E15		
44					15		1.46E7		3.11E15		
45					20		4220000		6.53E15		
46					5		3.75E8		6.37E16		
47		$(EO)_3P_{18}A$_Co			10		3.35E8		1.1E17		
48				3	15		3.66E8		1.1E17		
49					20		4.27E8		1.24E17		
50					5		4.79E7		1.71E16		
51		$(EO)_3P_{52}A$_Co			10		4340000		5.74E16		
52					15		4.01E7		1.53E15		12700
53					20		4620000		3.5E16		

Table S3: Summary of secondary structures elucidated from CD spectra using BeStSel (Beta Structure Selection).[7]

Entry		Peptide	Lipid	Embedded Polymer name	Polymer incorporation in mol %	α-helix	Anti-parallel β-sheets	Parallel β-sheets	Turn	Irregular / loop
**1**	$A\beta_{1-40}$ before aggregation					87.1	6.1	0	0	6.1
**2**	$A\beta_{1-40}$ after aggregation					0	0	100	0	0
**3**	POPC before aggregation	$A\beta_{1-40}$				0	50	0	50	0
**4**	POPC after aggregation	$A\beta_{1-40}$				5.8	5.8	0	82.6	0
**5**					5	6.1	87.7	0	0	6.1
**6**				$(EO)_2P_{19}A$_**Hy**	10	6.1	87.7	0	0	6.1
**7**					15	33.3	33.3	0	0	33.3
**8**					20	6.1	87.7	0	0	6.1
**9**					5	6.1	87.7	0	0	6.1
**10**				$(EO)_2P_{39}A$_**Hy**	10	6.1	87.7	0	0	6.1
**11**					15	6.1	87.7	0	0	6.1
**12**					20	6.1	67.7	0	0	6.1
**13**					5	87.7	6.1	0	0	6.1
**14**				$(EO)_3P_{12}A$_**Hy**	10	87.7	6.1	0	0	6.1
**15**					15	87.7	6.1	0	0	6.1
**16**					20	87.7	6.1	0	0	6.1
**17**					5	87.7	6.1	0	0	6.1
**18**				$(EO)_3P_{26}A$_**Hy**	10	87.7	6.1	0	0	6.1
**19**					15	87.7	6.1	0	0	6.1
**20**					20	76.4	11.8	0	0	11.8
**21**					5	87.7	6.1	0	0	6.1
**22**				$(EO)_2P_{22}A$_**Gl**	10	87.7	6.1	0	0	6.1
**23**					15	6.1	87.7	0	0	6.1
**24**					20	6.1	87.7	0	0	6.1
**25**					5	6.1	87.7	0	0	6.1
**26**				$(EO)_2P_{44}A$_**Gl**	10	6.1	87.7	0	0	6.1
**27**					15	6.1	87.7	0	0	6.1
**28**					20	6.1	87.7	0	0	6.1
**29**					5	6.1	87.7	0	0	6.1
**30**		$A\beta_{1-40}$	POPC	$(EO)_3P_{11}A$_**Gl**	10	6.1	87.7	0	0	6.1
**31**					15	33.3	33.3	0	0	33.3
**32**					20	33.3	33.3	0	0	33.3
**33**					5	87.7	6.1	0	0	6.1
**34**				$(EO)_3P_{42}A$_**Gl**	10	93.5	0	0	0	6.5
**35**					15	87.7	6.1	0	0	6.1
**36**					20	87.7	6.1	0	0	6.1
**37**					5	6.1	87.7	0	0	6.1
**38**				$(EO)_2P_{23}A$_**Co**	10	6.1	87.7	0	0	6.1
**39**					15	6.1	87.7	0	0	6.1
**40**					20	6.1	87.7	0	0	6.1
**41**					5	33.3	33.3	0	0	33.3
**42**				$(EO)_2P_{48}A$_**Co**	10	33.3	33.3	0	0	33.3
**43**					15	6.1	87.7	0	0	6.1
**44**					20	6.1	87.7	0	0	6.1
**45**					5	33.3	33.3	0	0	33.3
**46**				$(EO)_3P_{10}A$_**Co**	10	87.7	6.1	0	0	6.1
**47**					15	87.7	6.1	0	0	6.1
**48**					20	87.7	6.1	0	0	6.1
**49**					5	6.1	87.7	0	0	6.1
**50**				$(EO)_3P_{18}A$_**Co**	10	6.1	87.7	0	0	6.1
**51**					15	6.1	87.7	0	0	6.1
**52**					20	6.1	87.7	0	0	6.1
**53**					5	6.1	87.7	0	0	6.1
**54**				$(EO)_3P_{\cdot 52}A$_**Co**	10	6.1	87.7	0	0	6.1
**55**					15	6.1	87.7	0	0	6.1
					20	6.1	87.7	0	0	6.1

References

1. N. Sen, G. Hause and W. H. Binder, *Macromol Rapid Commun*, 2021, **42**, e2100120.
2. M. S. Terakawa, H. Yagi, M. Adachi, Y. H. Lee and Y. Goto, *J Biol Chem*, 2015, **290**, 815-826.
3. J. Habchi, S. Chia, C. Galvagnion, T. C. T. Michaels, M. M. J. Bellaiche, F. S. Ruggeri, M. Sanguanini, I. Idini, J. R. Kumita, E. Sparr, S. Linse, C. M. Dobson, T. P. J. Knowles and M. Vendruscolo, *Nat Chem*, 2018, **10**, 673-683.
4. N. Sen, C. Haupt, G. Hause, K. Bacia and W. H. Binder, *Macromol Biosci*, 2023, **23**, e2200522.
5. Z. Evgrafova, B. Voigt, A. H. Roos, G. Hause, D. Hinderberger, J. Balbach and W. H. Binder, *Phys. Chem. Chem. Phys.*, 2019, **21**, 20999-21006.
6. G. Meisl, J. B. Kirkegaard, P. Arosio, T. C. T. Michaels, M. Vendruscolo, C. M. Dobson, S. Linse and T. P. J. Knowles, *Nature Protocols*, 2016, **11**, 252-272.
7. A. Micsonai, F. Wien, L. Kernya, Y. H. Lee, Y. Goto, M. Refregiers and J. Kardos, *Proc. Natl. Acad. Sci.* , 2015, **112**, E3095-3103.
